# Punicalagin, a pomegranate polyphenol sensitizes the activity of antibiotics against three MDR pathogens of the *Enterobacteriaceae*

**DOI:** 10.1186/s12906-024-04376-7

**Published:** 2024-02-16

**Authors:** Saba Kiran, Anam Tariq, Shoaib Iqbal, Zubera Naseem, Waqar Siddique, Sobia Jabeen, Rizwan Bashir, Ashfaq Hussain, Moazur Rahman, Fazal-e Habib, Waqar Rauf, Aamir Ali, Yasra Sarwar, Georg Jander, Mazhar Iqbal

**Affiliations:** 1 Health Biotechnology Division, National Institute for Biotechnology and Genetic Engineering College, Pakistan Institute of Engineering and Applied Sciences (NIBGE-C, PIEAS), Faisalabad, Punjab 38000 Pakistan; 2 School of Biological Sciences, University of the Punjab, Quaid-i-Azam Campus, Lahore, Punjab Pakistan; 3 Boyce Thompson Institute, Cornell University, 14850 Ithaca, New York, USA

**Keywords:** MDR *Enterobacteriaceae,* Kandhari *P. granatum*, Punicalagin-antimicrobial synergism

## Abstract

**Background:**

Multidrug resistance (MDR) in the family *Enterobacteriaceae* is a perniciously increasing threat to global health security. The discovery of new antimicrobials having the reversing drug resistance potential may contribute to augment and revive the antibiotic arsenal in hand. This study aimed to explore the anti-*Enterobacteriaceae* capability of bioactive polyphenols from *Punica granatum* (*P. granatum*) and their co-action with antibiotics against clinical isolates of *Enterobacteriaceae* predominantly prevalent in South Asian countries.

**Methods:**

The Kandhari *P. granatum* (Pakistani origin) extracts were tested for anti-*Enterobacteriaceae* activity by agar well diffusion assay against MDR *Salmonella enterica* serovar Typhi*,* serovar Typhimurium and *Escherichia coli*. Predominant compounds of active extract were determined by mass spectrometry and screened for bioactivity by agar well diffusion and minimum inhibitory concentration (MIC) assay. The active punicalagin was further evaluated at sub-inhibitory concentrations (SICs) for coactivity with nine conventional antimicrobials using a disc diffusion assay followed by time-kill experiments that proceeded with SICs of punicalagin and antimicrobials.

**Results:**

Among all *P. granatum* crude extracts, pomegranate peel methanol extract showed the largest inhibition zones of 25, 22 and 19 mm, and the MICs as 3.9, 7.8 and 7.8 mg/mL for *S. typhi, S. typhi*murium and *E. coli,* respectively. Punicalagin and ellagic acid were determined as predominant compounds by mass spectrometry. In plate assay, punicalagin (10 mg/mL) was active with hazy inhibition zones of 17, 14, and 13 mm against *S. typhi, S. typhi*murium and *E. coli,* respectively. However, in broth dilution assay punicalagin showed no MIC up to 10 mg/mL. The SICs 30 μg, 100 μg, and 500 μg of punicalagin combined with antimicrobials i.e., aminoglycoside, *β*-lactam, and fluoroquinolone act in synergy against MDR strains with % increase in inhibition zone values varying from 3.4 ± 2.7% to 73.8 ± 8.4%. In time-kill curves, a significant decrease in cell density was observed with the SICs of antimicrobials/punicalagin (0.03–60 μg/mL/30, 100, 500 μg/mL of punicalagin) combinations.

**Conclusions:**

The *P. granatum* peel methanol extract exhibited antimicrobial activity against MDR *Enterobacteriaceae* pathogens. Punicalagin, the bacteriostatic flavonoid act as a concentration-dependent sensitizing agent for antimicrobials against *Enterobacteriaceae*. Our findings for the therapeutic punicalagin-antimicrobial combination prompt further evaluation of punicalagin as a potent activator for drugs, which otherwise remain less or inactive against MDR strains.

**Supplementary Information:**

The online version contains supplementary material available at 10.1186/s12906-024-04376-7.

## Background

South Asian countries including Pakistan are considered a hot zone for the fast-growing emergence of both multi-drug resistant (MDR) and extensively drug-resistant bacterial strains of the *Enterobacteriaceae* family [1, 2]. Resistance to last-resort antibiotics like carbapenem [3, 4], fluoroquinolones [5], and cephalosporin [6] have knocked down all health-related assurances by increasing the risk of morbidity and mortality rates associated with *Enterobacteriaceae* infections, making this an issue of significant global concern. For the upcoming two decades, a budget of approximately 100 trillion United States Dollars and millions of lives per year are considered at risk due to the emergence of “antibiotic resistance superbugs” with even worse consequences in low-middle income countries. More than 4.95 million mortalities are associated with increasing drug resistance against broad-spectrum antibiotics [7]. Several epidemiological reports all over the world proved the prevalence of multi-drug resistant extended-spectrum beta-lactamase-producing isolates belonging to *Enterobacteriaceae* from healthcare facilities as well as in community-acquired infections [8–10].

In the last decade, *Escherichia coli* (*E. coli)* has emerged as a microbe acquiring antibiotic resistance at an alarming rate with urinary tract infections being the most reported clinical infections in Pakistan and about 28 studies have described its high resistance against first-line antibiotics [11]. World Health Organization (WHO) has reported that *E. coli* resistance to third-generation cephalosporins and fluoroquinolones would lead to the end of the antibiotic era [12].

*Salmonella enterica* subspecies *enterica* serovar Typhi (*S. typhi*) causes the deadly systemic infection called typhoid fever also referred to as “enteric fever” when combined with paratyphoid fever caused by Paratyphi A, B and C [13]. Rapidly reducing susceptibility to fluoroquinolone in South Asian countries has been reported [14], which makes third-generation cephalosporins and azithromycin the drugs of choice. Although cephalosporin resistance has not been reported as extensively as fluoroquinolone resistance, *S. typhi* is acquiring resistance due to the production of extended-spectrum beta-lactamases, especially in Asian countries including Pakistan [15]. Every year 11.9 million to 27.1 million people, mostly children and elderly, suffer from enteric fever globally and mortality ranges from 129,000 to 223,000 [16].

*Salmonella enterica* serovar Typhimurium (*S. typhi*murium), a zoonotic serovar, is becoming a global threat due to its high antibiotic resistance rate in the last decade. Gastroenteritis is the major infection caused by non-typhoidal *Salmonella* in humans with 80.3 million foodborne illnesses per year [17]. A study reported that 35.2% of isolates of *S. typhi*murium were carrying the beta-lactam (carbapenems and cephalosporins) resistance gene (*bla*_TEM-1_) in Pakistan [1].

Overall, in the *Enterobacteriaceae*, cephalosporin resistance is due to the production of enzymes called beta-lactamases such as extended-spectrum beta-lactamase, AmpC beta-lactamases [18] and quinolone resistance is due to gene mutation in quinolone resistance-associated genes [19]. This resistance can be correlated with the irrational use of antibiotics in food animals for their better growth and infection prevention, which makes a linkage of antibiotic resistance between food and its consumers i.e., animals and humans [20]. This rapid emergence of antibiotic resistance warrants re-screening of natural products as a faster approach to identify new “antimicrobial magic bullets”, which can act alone or contribute synergistic effects with the inactive or lesser active existing antibiotics to augment their potencies and reverse their drug resistance. In this manner, exploration of phytochemicals may contribute to identify novel antibiotic potentiators to augment the potencies of drugs against which the resistance has already emerged and/or the first-line antibiotics, which will likely face resistance in near future [21–24].

*Punica granatum* is famous due to its medicinal potential [25], which is rich in metabolites having anticancer, antimicrobial and antidiabetic potential [26]. Both the edible fruit and non-edible (peels, seeds, flowers, leaves and bark) parts of this plant have metabolites with substantial antimicrobial properties [27]. The major fraction of the fruit consists of the peel, which is mostly discarded as waste without any commercial utilization. The interesting fact is that peel extract has the highest amount of bioactive phenolic compounds such as ellagitannins, tannins [28] and anthocyanin [29, 30], including ellagic acid as well as punicalagin (2,3-hexahydroxydiphenoylgallagyl-D-glucose) [31]. Recently, pomegranate extracts were evaluated against beta-lactamase-producing drug resistance Gram-positive and Gram-negative bacteria using agar diffusion and minimum inhibitory concentration assays [27, 32].

In the current study, we compared the anti-*Enterobacteriaceae* activity of different solvent extracts of edible and non-edible parts of Kandhari pomegranate of Pakistan. Because of the remarkable antibacterial activity of *P. granatum* peel methanol extract, it was further investigated by mass spectrometric analysis that revealed punicalagin as a major constituent of pomegranate extract. Punicalagin was further explored for its bioactivity and as an antimicrobial adjuvant/potentiator efficacy in conjunction with the representatives of different classes of antimicrobials against the MDR *Enterobacteriaceae*.

## Methods

### Pomegranate peel powder

*P. granatum* peels (50 kg), obtained as a co-product during pomegranate juice extraction, were supplied by a local juice shop located in Jinnah Market (Faisalabad). The collected peels were then rinsed with distilled water. The peels were air-dried under ambient conditions and maintained at − 20 °C in vacuum-sealed packages. A grinder mill and sieves were used to obtain a powder particle size of less than 0.417 mm.

### Pomegranate whole fruit juice, pomegranate fresh seed juice and pomegranate dried seed powder

*P. granatum* fruits were freshly procured from a local market and divided into two portions. From the one portion pomegranate whole fruit juice was obtained by pressure extraction of the whole fruit (15 units of fruit that weighed 8 kg). After chopping with a grinder, pomegranate pieces were ground and juice was sieved. From the other portion, the edible seeds were separated from the peels and the total weight of the arils was divided into two equal parts. Pomegranate fresh seed juice was obtained by pressure extraction of the fresh arils. While the other portion was dried under shade to get pomegranate dried seed powder. A grinder mill and sieves were used to obtain a powder particle size of less than 0.417 mm. Liquid samples were dried by a rotary evaporator. All the prepared samples were stored at − 20 °C in vacuum-sealed packages until analysis (2 months as a maximum).

### Preparation of extracts

An amount of 200 g of each of the samples of pomegranate was separately blended (using a blender for 2 minutes) with solvents having an increasing polarity: 100% ethyl acetate, 80% methanol, 100% methanol, 70% ethanol, 100% ethanol, 100% water and boiling in 100% water. Dilutions for the varying concentrations were accomplished using distilled water. The samples were incubated at 37 °C for 2–8 h in a shaking incubator (Witeg Wisd shaking incubator WIS20, Germany) at 200 rpm. After this, the sample extracts were filtered with Whatman No. 1 filter paper in a Buchner funnel to remove peel particles and concentrated under reduced pressure at 40 °C in a rotary Evaporator (Heidolph, Schwabach Germany) to remove almost 90% of the solvent. It was further dried in a desiccator under a vacuum to achieve constant weight [33]. The extraction process was repeated three times to extract the maximum components from each sample. Dried extracts were dissolved in 100 and 30% liquid chromatography-mass spectrometry grade (LC-MS) methanol for MS analysis and antimicrobial assay, respectively.

### Yield of extract

The yields of all extracts (extractable components) expressed on a dry weight basis were calculated from the following equation and reported as percent yield.

$$\% Yield\ \left(\frac{g}{100\ g}\right)=\frac{W1\times 100}{W2}$$

where W1 is the weight of the extract residue obtained after concentration and drying, whereas, W2 is the weight of the peel or pulp taken [33].

### Bacterial strains

A total of 3 Gram-negative MDR clinical isolates including *S. typhi*, *S. typhi*murium and *E. coli* were used to screen for the antimicrobial activity of all the prepared extracts of pomegranate. All isolates used in this study were clinical, and isolated from hospitalized patients (Allied Hospital, Faisalabad, Pakistan). *S. typhi* and S. Typimurium were from typhoid-suspected patients and *E. coli* was from UTIs suspected patients, originally collected and stored by NIBGE bacterial stock culture department. All the clinical isolates were identified by staining characters and morphology; colony characters and pigmentation and reaction in triple sugar iron agar media. For molecular confirmation of the isolates, genus-specific PCR was performed using previously reported protocols [34] for *Salmonella* and *E. coli* [35]. Genomic DNA extraction was done by the chloroform-isoamyl alcohol method [36]. A highly specific *stm* gene fragment was selected for the identification of serovar Typhimurium [37], *fliC* for serovar Typhi and *uidA* gene for *E. coli* [35]. A list of the primers used in the identifications of isolates is given in Table S1. The final products were confirmed with 1.5% agarose gel electrophoresis.

### Determination of the antimicrobial resistance profile of the *Enterobacteriaceae* strains

Antimicrobial susceptibility studies were carried out on the bacterial isolates using commercially available antimicrobial discs (Oxoid) by the Kirby Bauer disc diffusion technique. Lauria-Bartani (LB) broth (Himedia, Mumbai, India) containing 0.5 McFarland (MF) turbidity (0.14–0.17 OD_600_) of bacterial culture was swabbed on Mueller Hinton agar plates. Antimicrobial discs were placed on prepared plates about 20 mm apart, inhibition zone diameter was measured after 16–18 h incubation at 37 °C, and the results were interpreted as per Clinical and Laboratory Standard Institute guidelines [38]. Antimicrobial discs from seven major antimicrobial groups were included for phenotypic susceptibility testing as given in Table 1.

**Table 1:** Phenotypic antimicrobial resistance profiles of clinical isolates used in current study

Sr. No.	Antimicrobials	Code-Disc potency (μg)	*S. typhi*murium	*S. typhi*	*E. coli*.
1	Sulfamethoxazole /Trimethoprim	SXT-23.75/1.25	R	R	R
2	Nalidixic acid	NA-30	R	R	R
3	Ampicillin	AMP-10	S	S	R
4	Chloramphenicol	C-30	S	S	R
5	Aztreonam	ATM-30	IR	IR	IR
6	Amoxicillin/ Clavulanic acid	AMC-30	IR	S	IR
7	Gentamicin	GEN-10	S	R	R
8	Ceftriaxone	CRO-30	IR	IR	R
9	Ciprofloxacin	CIP-5	R	S	R

*R* Resistant: *IR* Intermediate resistant: *S* Sensitive

### Mass spectrometric analysis of the crude extracts

The detailed investigation of crude extracts was completed using a mass Spectrometer (LTQ XL Linear Ion Trap, Thermo Fisher Scientific, Waltham, MA, United States), furnished with an Electrospray Ionization probe. Approximately 5 mg of the extract was dissolved in 5 mL methanol (LC-MS grade), which was further diluted 10 times with methanol. The sample after passing through the polytetrafluoroethylene filter membrane (0.45 μm) was injected into the mass spectrometer using a direct syringe pump with a flow rate of 10 μL min^−1^. The sample was analyzed on positive and negative ionization modes within the range of *m/z* 50–2000. The capillary and source voltages were tuned at 35 kV and 4.2 kV, respectively, for positive ion mode and -30 kV and − 4.5 kV, respectively, for negative ion mode. Capillary temperature (280 °C), nitrogen flow rate (25 L.min^−1^), and auxiliary gas flow rate (5 L min^−1^) were set at positive and negative ion modes for full scan and MS^2^. The ion peaks were further fragmented using Collision-Induced Dissociation. The MS and MS^2^ data were obtained and processed using Xcalibur software. The chemical structures of parent and daughter ion peaks were drawn using ChemDraw Ultra 12.0 software. The identification of compounds was confirmed by their fingerprinting fragments with reference standards and literature values. The Mass Spectrometry analysis was performed as described by Mphahlele et al. [39] and pure reference standards (Sigma-Aldrich, Germany) were used to confirm the presence or absence of ellagic acid (Sigma-Aldrich, CAS No. 476–66-4) and punicalagin (MedChem Express, Cat. No. HY-N0063, > 99.97%.

### In vitro antimicrobial activity of extracts

The agar well diffusion assay, similar to that reported previously [40], was conducted to evaluate the inhibitory spectrum of the extracts and pure compounds (ellagic acid, punicalagin), selected after mass spectrometry against test microorganisms (Table 2). Freshly grown bacterial culture [70 μl) in Mueller Hinton broth was adjusted to the final inoculum density of 10^7^ cfu/mL (by 0.5 MF), spread on agar plates and left to get dried for 30 min. The wells (6 mm in diameter) were made in media using a sterilized stainless steel borer. Each well was filled with 100 μL (700 mg/mL) of diluted extracts. The plates were left at room temperature for 30 min to allow the diffusion of materials in the media. The methanol was used as the vehicle control. Antibacterial activity was expressed as the diameters of the zone of inhibition (ZOI) produced around each well measured after incubation time. The plates were incubated at 37 °C for 16–18 hrs. The experiment was repeated three times to confirm the reproducibility of the observed data.

**Table 2:** Percentage yield and antimicrobial activity of *Punica granatum* extracts and pure compounds against clinical isolates

Sr. No.		Solvent	Concentration % (v/v)	Extract Yield (%)	*S. typhi*murium	*S. typhi*	*E. coli*
	**Extract**						
1	**PPE**	Methanol	100	8.06	13.0	11.0	13.0
2			80	10.12	18.0	20.0	20.0
3		Ethanol	100	9.0	14.0	13.0	11.0
4			70	11.5	16.0.	17.0	17.0
5		Ethyl acetate	100	0.94	7.0	7.0	7.0
6		Distilled H_2_O	100	8.10	11.0	9.0	9.0
7		Distilled H_2_O with boiling	100	8.57	11.0	9.0	11.0
8	**PDSE**	Methanol	100	11.2	9.0	9.0	9.0
9			80	15.3	11.0	11.0	11.0
10		Ethanol	100	9.00	9.0	9.0	9.0
11			70	12.5	10.0	10.0	10.0
12		Ethyl acetate	100	1.66	7.0	7.0	7.0
13		Distilled H_2_O	100	13.5	9.0	9.0	9.0
14		Distilled H_2_O with boiling	100	13.45	9.0	9.0	9.0
15	**PWFE**	Methanol	100	8.49	13.0	13.0	11.0
16			80	10.3	17.0	15.0	15.0
17		Ethanol	100	8.04	11.0	11.0	11.0
18			70	9.45	13.0	13.0	13.0
19		Ethyl acetate	100	0.51	7.0	7.0	7.0
20		Distilled H_2_O	100	9.29	11.0	10.0	10.0
21		Distilled H_2_O with boiling	100	9.18	11.0	11.0	9.0
22	**PFSE**	Methanol	100	11.3	9.0	9.0	9.0
23			80	17.6	13.0	13.0	12.0
24		Ethanol	100	9.05	9.0	9.0	9.0
25			80	19.2	11.0	11.0	10.0
26		Ethyl acetate	100	0.73	7.0	7.0	7.0
27		Distilled H_2_O	100	8.97	9.0	9.0	9.0
28		Distilled H_2_O with boiling	100	8.67	9.0	9.0	9.0
	**Pure compounds**						
29	Ellagic acid			–	0.0	0.0	0.0
30	Punicalagin			–	14(Hazy)	17(Hazy)	13(Hazy)

*PPE* pomegranate peel extract: *PDSP* Pomegranate dried seed extract: *PWFE* Pomegranate whole fruit extract: *PFSE* pomegranate fresh seed extract

### Determination of minimum inhibitory concentration

The MIC values of peel extract in methanol and punicalagin against clinical isolates were determined by the standard method of broth micro-dilution, to find the lowest concentration at which no visible growth of the bacteria was observed. Briefly, a two-fold serial dilution of methanol peel extract stock (700 mg/mL) was made to acquire 10 concentrations in the range of 350 to 0.6 mg/mL and punicalagin (20 mg/mL) was diluted to prepare the concentrations in the range of 10 to 0.25 μg/mL. In the 96-well microtitre plate, one row of 12 wells contained only LB (200 μL/well) as a ‘blank’, while another row contained only methanol (200 μL/well) as the ‘vehicle control’ serially diluted (with LB; v/v) to the concentration corresponding to the respective wells used for MIC determination of peel methanol extract. The broth culture containing 0.5 MF (1 × 10^7^ cfu/mL) inoculum density was introduced to each of the wells, except the ‘sterility control lane’ (blank lane), at a 1:10 ratio to maintain a final inoculum density of 1× 10^7^ cfu/mL. After incubation for 22 h at 37 °C, the bacterial growth was determined at 600 nm using ELISA Reader (Synergy H1 Biotek Microplate Reader). The optical density of the ‘vehicle control’ lane indicated the maximum growth of the test bacteria, while the ‘blank’ lane showing no growth served as a ‘sterility control’ for the procedure. The MIC value is defined as the lowest concentration of the compound that will inhibit the visible growth of a microorganism after overnight incubation [41].

### Determination of antibacterial synergy of antibiotics with punicalagin by agar disc diffusion assay

Agar disc diffusion assay was used for initial combination experiments against three clinical strains *S. typhi, S. typhi*murium and *E. coli.* Mueller Hinton agar plates were prepared by spreading the bacterial growth of 0.5 MF using cotton swabs dipped in the 0.5 MF followed by the placement of antimicrobial discs. Punicalagin stock solutions of 20 mg/mL were prepared and applied on antimicrobial discs at sub-inhibitory concentrations of 30 μg/antimicrobial disc, 100 μg/antimicrobial disc, and 500 μg/antimicrobial disc. Overnight incubation of 37 °C followed by ZOI measurement and percentage (%) increase in inhibition zone was calculated as (b^2^-a^2^)/a^2^ × 100, where “a” is the inhibition zone of antibiotic alone and “b” is the antibiotic plus punicalagin zone. A combination assay was performed in triplicate and the standard deviation was calculated. All values are expressed as the mean standard error (±) of the mean of triplicate values of the same replicate [42]. Statistical comparisons on combination effects by disc diffusion method were performed using a Student‘s *t*-test by Tukey posthoc test. A *P*-value of < 0.05 was considered statistically significant. The synergistic combinations resulting in > 30% inhibition were confirmed by plotting the time-kill curve [43].

## Results

### Extraction yield

**Fig. 1:**
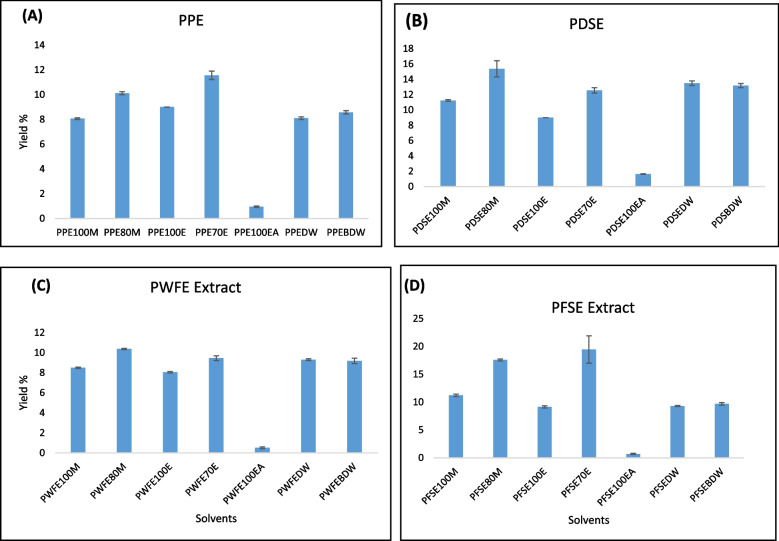
Comparative yields of pomegranate extracts in different solvents. (**A**) Pomegranate peel powder extract, (**B**) Pomegranate dried seed powder extract, (**C**) Pomegranate whole fruit juice extract, and (**D**) Pomegranate fresh seed juice extract. 100 M: 100% methanol, 80 M: 80% methanol, 100E: 100% ethanol, 70E: 70% ethanol, 100EA: 100% ethyl acetate, DW: distilled water, BDW: distilled water with boiling

The average yields of all extracts obtained with different solvents are given in Table 2 and Fig. 1. For pomegranate peel extraction, the total yield was higher in 80% ethanol (11.5%) than in any used concentrations of methanol, ethyl acetate and water (Table 2, Sr. 1–7). For dry seed extraction, the highest (15.3%) and the lowest yields (1.66%) were obtained with 80% methanol and 100% ethyl acetate, respectively (Table 2, Sr. 8–14). Pomegranate whole fruit juice extract gave a yield between the range of 0.51% with ethyl acetate to 10.3% with 80% methanol (Table 2, Sr. 15–21) and 0.73% with ethyl acetate to 19.2% with 80% ethanol for the fresh seed extract (Table 2, Sr. 21–28).

### Biochemical and molecular confirmation of clinical isolates

Clinical isolates taken from NIBGE stock cultures were confirmed by Gram staining resulting in small red Gram-negative rods visible in microscopic view. A characteristic yellow butt of the test tube with a pink slant showing a black center due to H_2_S production in the Tryptic soy agar slant was a typical confirmation of *Salmonella* spp. Lactose fermenting (pink) colonies on MacCkonkey agar and yellow slant and yellow butt with gas formation but no H_2_S in the TSI agar slant was characteristic of *E. coli*.

Molecular confirmation of genus *Salmonella* was done using *invA* gene fragment (284 bp amplification), and the serovars were confirmed by targeting gene fragments: *stm* (401 bp amplification) for *S. typhi*murium, *fliC* (495 bp amplification) for *S. typhi* and *uidA* (486 bp amplification) for *E. coli.* All oligonucleotides used for confirmation are given in **(**supplementary materials, Table S1**)**.

### Antibiotic resistance profiling

The isolates resistant to at least three different classes of antibiotics were considered as MDR. The resistance profile of each isolate is compiled in **(**Table 1). All clinical isolates used in this study were found to be MDR based on their antibiotic resistance profiles. The ciprofloxacin-resistant *E. coli* and *S. typhi*murium and ceftriaxone-resistant *S. typhi* were proceeded for further evaluation of synergy interaction of resistant antibiotics with most active peel methanol extract.

### Mass spectrometric analysis of pomegranate extract

**Fig. 2:**
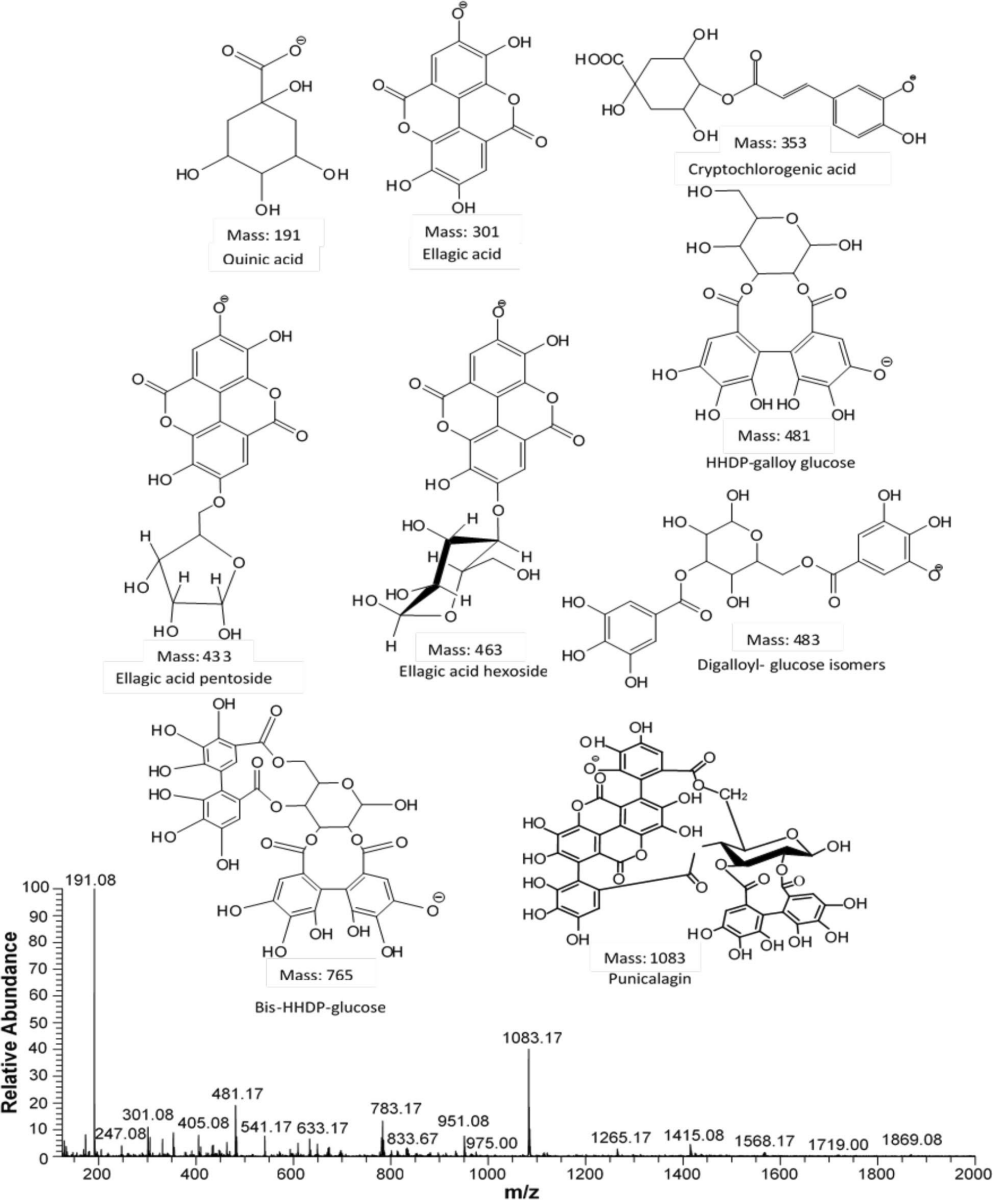
ESI-MS/MS analysis of *Punica granatum* peel powder methanol extract in negative ion mode

The ESI-MS^n^ method was used to identify the predominant compounds in crude extracts. The full scan mass spectrum of pomegranate peel methanol extract at negative ion mode (*m/z* 100–1500) showed the presence of molecular ions [M-H]^−^ of quinic acid (*m/z 191,* 100% abundance), ellagic acid (*m/z 301*, 14% abundance), cryptochlorogenic acid (*m/z 353,* 10% abundance), ellagic acid pentoside (*m/z 433,* 26% abundance), ellagic acid hexoside (*m/z 463,* 1.5% abundance), a fragment of pedunculagin *m/z 481,* 20% abundance), digalloyl-glucose isomer (*m/z 483,* 9% abundance), Pedunculagin (*m/z 783,* 15% abundance) and punicalagin (*m/z 1083,* 38% abundance) (Fig. 2).

The molecular ion peak of quinic acid (*m/z* 191) was subjected to MS^2^ fragmentation to generate daughter ion peaks for comprehensive analysis (Supplementary Materials, Fig. S1A). A fragmented peak with *m/z* 173 was produced by the removal of the H_2_O molecule from which further ion peaks were generated at *m/z* 129 and *m/z* 155 by the loss of CO_2_ and H_2_O molecules, respectively. The subsequent fragmentation of the ion at *m/z* 155 yielded a base ion peak at *m/z* 111 with approximately 100% relative abundance by the loss of CO_2._ The molecular ion peak of ellagic acid (*m/z* 301) generated two prominent ion peaks at *m/z* 283 and *m/z* 257 after losing one molecule of H_2_O and one molecule of CO_2_, respectively (Supplementary Materials, Fig. S1B). The peak with m/z 257 further split into the fragment ions at *m/z* 213 and *m/z* 229 with the removal of CO_2_ and subsequent H_2_O. Cryptochlorogenic acid at (*m/z* 353) generated three daughter ions at *m/z* 293, m/z 265, and *m/z* 247 with the loss of CO_2_, CO and H_2_O molecules, respectively (Supplementary Materials, Fig. S1C). Ellagic acid pentoside (*m/z* 433) fragmented into two daughter ions with *m/z* of 301 (ellagic acid) and 153 (Supplementary Materials, Fig. S1D). Ellagic acid hexoside (*m/z* 463) showed the most abundant fragment ion at *m/z* 301 by the loss of hexoside sugar (Supplementary Materials, Fig. S1E). The fragment of HHDP-Galloy glucose showed a molecular ion peak at *m/z* 481 that further fragmented into the ions at *m/z* 301 (ellagic acid) by losing glucose molecule and at *m/z* 275 by losing CO from ellagic acid, respectively (Supplementary Materials, Fig. S1F). The fragment ion peak at *m/z* 463 was also observed by the loss of H_2_O molecule from the fragment of pedunculagin.

A digalloyl-glucose isomer was characterized by a molecular ion [M-H]^−^ at *m/z 483* that fragmented into daughter ions at *m/z 451*, *m/z 465* and *m/z 439* representing [M-H-O_2_]^−^, [M-H-H_2_O]^−^ and [M-H-CO_2_]^−^, respectively, and also at *m/z 331* and *m/z 169* (Supplementary Materials*,* Fig. S1G). The fragment at *m/z 331* further lost an H_2_O molecule generating an ion *m/z 313*. A pair of fragments attributed to cross-ring fragmentation appeared at *m/z 271* and *m/z 241.* The molecular ion of pedunculagin at *m/z 783* showed two peaks of dissociated ions at *m/z 765* and *m/z 481* and one ion peak at *m/z 633* with ring removal. A couple of other fragment peaks appeared at *m/z 301* and *m/z 275* in this spectrum (Supplementary Materials, Fig. S1H).

The molecular ion of punicalagin [M-H]^−^ appeared at *m/z 1083* that dissociated into the ion peak at *m/z* 1065 corresponding to [M-H-H_2_O]^−^ (Supplementary Material, Fig. S1I). The peak at *m/z 1065* split into another fragment at *m/z 763* showing the loss of the ellagic acid part that further generated the peak at *m/z 721* characterized by the concomitant loss of CO_2_. The punicalagin molecular ion peak also showed the fragmentation peaks at *m/z 781, m/z 601* and *m/z 575*.

### Comparative evaluation of the antibacterial activity of pomegranate extracts and pure compounds against drug-resistant clinical isolates

**Fig. 3:**
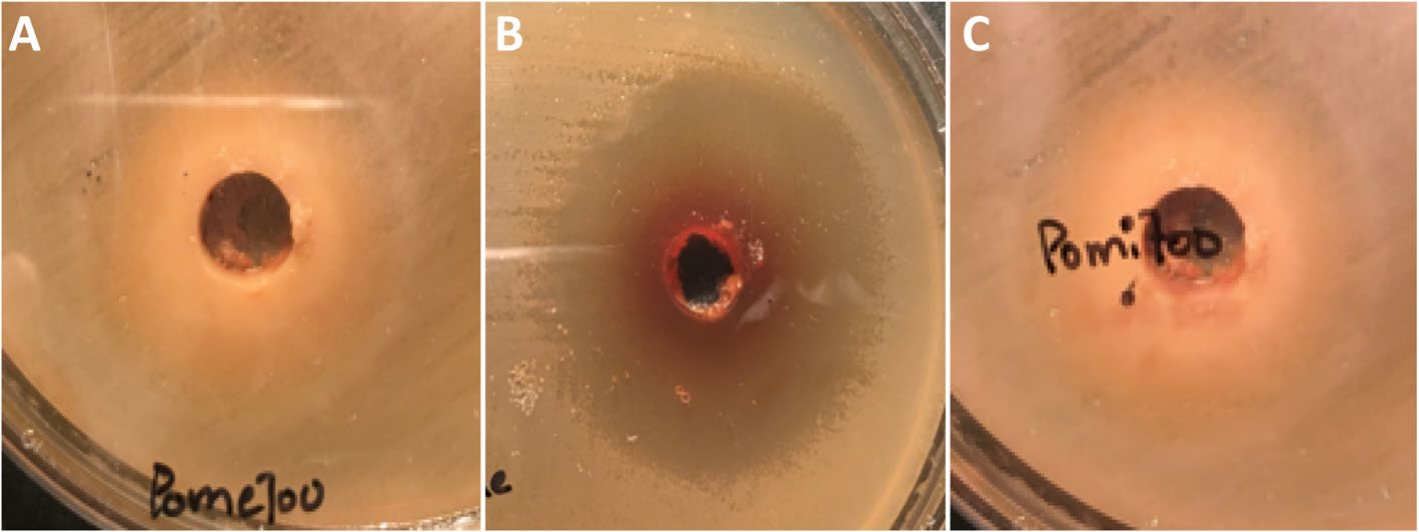
Growth inhibition zones of pomegranate peel methanol extract against MDR *Enterobacteriaceae* clinical isolates. **A** Growth inhibition zone (18 ± 1 mm) against *S. typhi*murium produced by pomegranate peel methanol extract (700 mg/mL) on Mueller Hinton agar plate, **B** Growth inhibition zone against *S. typhi* (20 ± 1 mm) pomegranate peel methanol extract (700 mg/mL) on Mueller Hinton agar plate, **C** Growth inhibition zone (20 ± 1 mm) against *E. coli* produced by pomegranate peel methanol extract (700 mg/mL) on Mueller Hinton agar plate

The antimicrobial activity of crude pomegranate extracts and pure compounds, evaluated by agar well diffusion assay, against selected strains is given in Table 2. It was evident from the results that pomegranate peel methanol extract exhibited the largest ZOI in comparison to all other extracts against *S. typhi*, *S. typhi*murium and *E. coli* followed by crude 70% ethanolic extract of pomegranate peel while *S. typhi* and *E. coli* showed equal sensitivity to the same concentration of pomegranate peel methanol extract, and *S. typhi*murium was found to be more resistant for the same concentration (Table 2, Serial No. 2, Fig. 3).

The most satisfactory results of pomegranate dried seed extract were also recorded with 80% methanol extract but the largest inhibition zone appeared against *S. typhi*murium (Table 2, Serial No. 9).

The trend was followed by pomegranate whole fruit juice methanol extract (Table 2, Serial No. 16) and pomegranate fresh seed methanol extract (Table 2, Serial No. 23), which demonstrated better antibacterial activity as compared to other solvents.

All the extracting solvents other than 80% methanol showed approximately equal inhibition zones against all pathogenic strains that were lower than inhibition zones produced by 80% methanol extracts **(**Table 2**)**. It means that hydroalcoholic solvent systems are the best for the extraction of bioactive secondary metabolites of pomegranate.

**Fig. 4:**
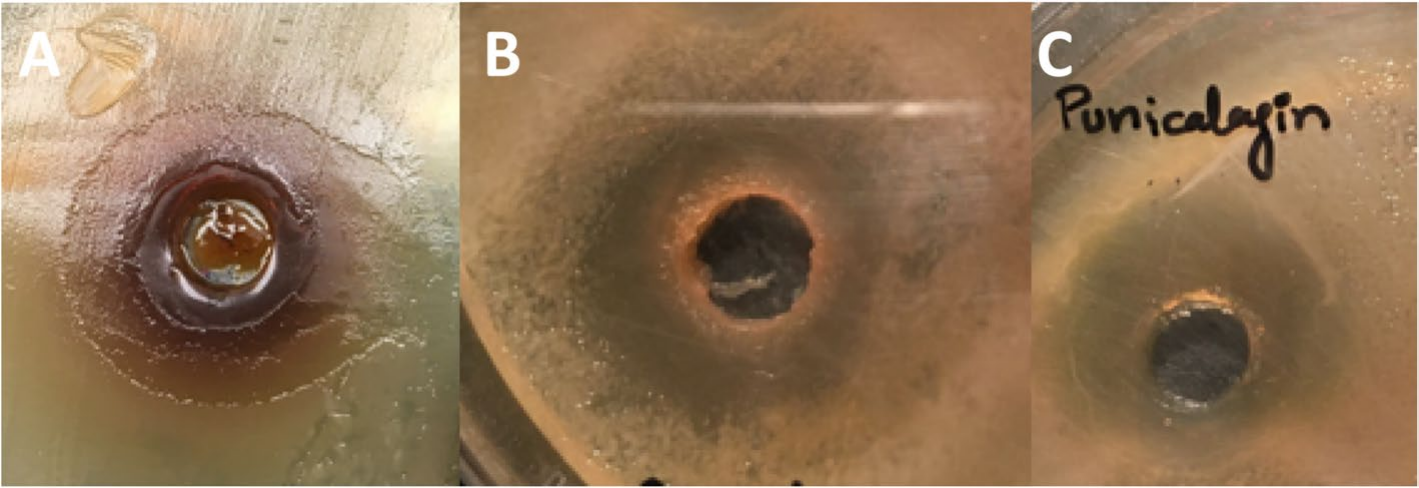
Growth inhibition zones of punicalagin against MDR *Enterobacteriaceae* clinical isolates. **A** Growth inhibition zone (17 mm, hazy) against *S. typhi* produced by punicalagin (20 mg/mL) on Mueller Hinton agar plate, **B** Growth inhibition zone against *S. typhi*murium (14 mm, hazy) produced by punicalagin (20 mg/mL) on Mueller Hinton agar plate, **C** Growth inhibition zone (13 mm, hazy) against *E. coli* produced by punicalagin (20 mg/mL) on Mueller Hinton agar plate

The overall inhibitory activity of pomegranate extracts against *Enterobacteriaceae* spp. observed in the current study followed the trend as 80% methanol > 70% ethanol > 100% methanol > 100% ethanol > distilled water with boiling > distilled water at room temperature > ethyl acetate. While among two of the tested pure compounds (ellagic acid and punicalagin), only punicalagin was found to be active showing hazy inhibition zones against MDR pathogens in well diffusion assay (Table 2, Serial No. 29–30, Fig. 4) and selected for further investigation.

### Determination of minimum inhibitory concentration and % inhibition

Minimum inhibitory concentration was evaluated by broth dilution assay in 96 well plates according to Clinical and Laboratory Standards Institute guidelines 2021 [38], which resulted in MIC values ranging from 3.9–7.8 mg/mL for pomegranate peel methanol extract against all the tested clinical strains while surprisingly punicalagin was unable to show MIC value even at a concentration of 10 mg/mL (Table 3**)**. This is why three minimum sub-inhibitory concentrations of punicalagin were chosen to determine the efficacy to potentiate the drugs. Although further investigations, i.e., synergistic disc diffusion assay and growth curve assay revealed the bacteriostatic nature of punicalagin.

**Table 3:** MIC of pomegranate peel methanol extract and punicalagin for MDR clinical strains of *Enterobacteriaceae*

Sr. No.	Clinical Strains	Minimum Inhibitory Concentration (mg/mL)	
		Pomegranate peel methanol extract	Punicalagin
1	*E. coli*	7.8	> 10
2	*S. typhi*	3.9	> 10
3	*S. typhi*murium	7.8	> 10

Vehicle control: Methanol (HPLC grade); Sterility control/blank: LB broth

The optical density of the ‘vehicle control’ lane indicated the maximum growth of the test bacteria, while the ‘blank’ lane showing no growth served as a ‘sterility control’ for the procedure

**Fig. 5:**
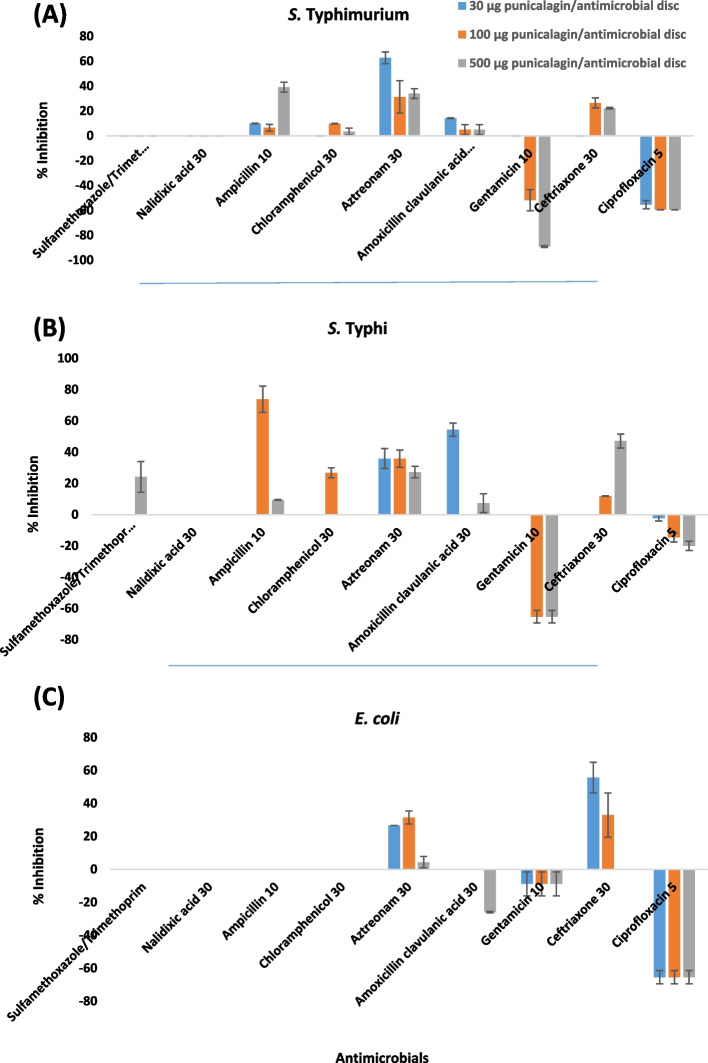
Combination effects of sub-inhibitory concentrations of punicalagin with the representatives of different classes of antimicrobials against MDR *Enterobacteriaceae* clinical isolates. **A** % increase in zone of inhibition resulted in a combination of 30 μg punicalagin/antimicrobial disc, 100 μg punicalagin/antimicrobial disc and 500 μg punicalagin/antimicrobial disc against *S. typhi*murium by agar disc diffusion assay, **B** % increase in zone of inhibition resulted in a combination of 30 μg punicalagin /antimicrobial disc, 100 μg punicalagin/antimicrobial disc and 500 μg punicalagin/antimicrobial disc against *S. typhi* by agar disc diffusion assay, **C** % increase in zone of inhibition resulted in a combination of 30 μg punicalagin/antimicrobial disc, 100 μg punicalagin/antimicrobial disc and 500 μg punicalagin/antimicrobial disc against *E. coli* by agar disc diffusion assay

**Fig. 6:**
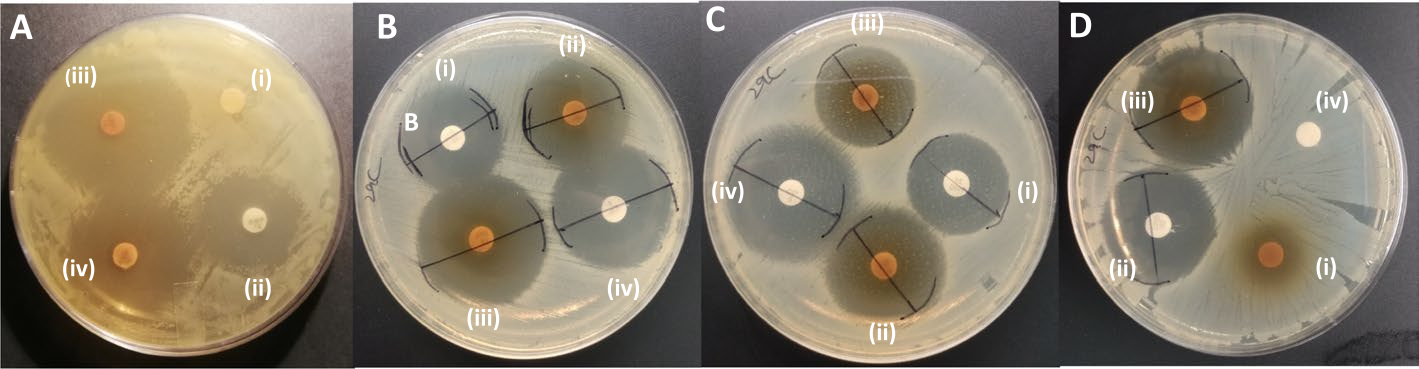
Combination effect of punicalagin with antimicrobials against *S. typhi*, (**A**) Combination effect of punicalagin with ampicillin (Amp-10) against MDR *S. typhi*, (i) 100 μg of punicalagin showing no inhibition zone, (ii) Inhibition zone produced by ampicillin without punicalagin, (iii) Synergistic inhibition zone produced by 100 μg punicalagin with ampicillin showing 73% increase in fold area, (iv) Synergistic Inhibition zone produced by 500 μg punicalagin with ampicillin showing 26% in fold area, (**B**) Combination effect of punicalagin with aztreonam (ATM-30) against MDR *S. typhi*, (i) Inhibition zone produced by aztreonam without punicalagin, (ii) Synergistic inhibition zone produced by 100 μg punicalagin with aztreonam showing 35% increase in fold area, (iii) Synergistic inhibition zone produced by 500 μg punicalagin with aztronam showing 47% increase in fold area, (iv) Ciprofloxacin inhibition zone as positive control, (**C**) Combination effect of punicalagin with amoxicillin clavulanic acid (AMC-30) against MDR *S. typhi*, (i) Inhibition zone produced by amoxicillin clavulanic acid without punicalagin, (ii) Synergistic Inhibition zone produced by amoxicillin clavulanic acid with 30 μg punicalagin showing 54% increase in fold area, (iii) Indifferent Inhibition zone produced by amoxicillin clavulanic acid with 100 μg punicalagin showing 0% increase in fold area, (iv) Ciprofloxacin inhibition zone as positive control, (**D**) Combination effect of punicalagin with ceftriaxone (CRO-30) against MDR *S. typhi*, (i) 500 μg of punicalagin showing no inhibition zone, (ii) Inhibition zone produced by ceftriaxone without punicalagin, (iii) Synergistic inhibition zone produced by 500 μg punicalagin with ceftriaxone showing 47% increase in fold area, (iv) Methanol showing no inhibition zone as negative control

In vitro activity evaluation of punicalagin combined with antibiotics by disc diffusion assay proved to be less laborious and easy to interpret with minimum error chances due to repetitive experiments. The combination effects of sub-inhibitory concentrations of punicalagin with the representatives of different classes of antimicrobials against MDR *Enterobacteriaceae* clinical isolates are given in Fig. 5. Sub-inhibitory concentrations of punicalagin as 30 μg, 100 μg and 500 μg per minimum inhibitory concentrations of antibiotics were used to evaluate the combination effects against all the tested isolates (Table 4). Sulfamethoxazole/Trimethoprim 23/1.25 μg showed no synergism with all tested concentrations of punicalagin against *S. typhi*murium and *E.coli* but combining with 500 μg of punicalagin increased the inhibition area to 63.8% against *S. typhi* (Table 4, Serial No. 1, Fig. S2). Nalidixic acid 30 μg demonstrated no augmenting efficacy with 30, 100 and 500 μg punicalagin for all three tested clinical isolates (Table 4, Serial No. 2). Combination of ampicillin 10 μg with 30 μg of punicalagin increases the inhibition fold area to 9.9% for *S. typhi*murium while further increment of punicalagin to 100 μg showed less augmenting potential (6.5%). Surprisingly 500 μg showed 39% increase in growth inohibition fold area for *S. typhi*murium (Table 4, Serial No. 3). For *S. typhi* 30 μg/10 μg ampicillin showed no synergism and highest combination activity of 73% was seen at the concentration of 100 μg/10 μg ampicillin (Table 4, Serial No.3, Fig. 6A), while 500 μg/10 μg ampicillin enhances the % inhibition fold area to only 9.4% (Table 4, Serial No. 3). However, the combinations ampicillin 10 μg/30, 100, 500 μg punicalagin remained indifferent for *E. coli* (Table 4, Serial No.3). Chloramphenicol 30 μg showed no synergistic growth inhibition with 30 μg of punicalagin but further concentrating punicalagin to 100 and 500 μg demonstrated synergism by increasing the fold area to 9.7 and 3.4% respectively for *S. typhi*murium (Table 4, Serial No.4). For *S. typhi* only the combination 100 μg punicalagin/chloramphenicol 30 μg showed 26% increase in inhibition fold area while 30 and 500 μg of punicalagin with 30 μg of chloramphenicol was proved in effective combinations to increase the inhibiting efficacy (Table 4, Serial No.4).

**Table 4:** Combination effects of sub-inhibitory concentrations of punicalagin combined with minimum inhibitory concentrations of antibiotics

Sr. No.	Antibiotics	Code (μg/disc)	Increase in fold area by adding punicalagin (30, 100, 500 μg/disc) (%)								
			*S. typhi*murium			*S. typhi*			*E. coli*		
			**30** μg/disc	**100** μg/disc	**500** μg/disc	**30** μg/disc	**100** μg/disc	**500** μg/disc	**30** μg/disc	**100** μg/disc	**500** μg/disc
1	**Sulfamethoxazole/Trimethoprim**	SXT 23.75/1.25	0 ± 0	0 ± 0	0 ± 0	0 ± 0	0 ± 0	63.8 ± 11.3	0 ± 0	0 ± 0	0 ± 0
2	**Nalidixic acid**	NA 30	0 ± 0	0 ± 0	0 ± 0	0 ± 0	0 ± 0	0 ± 0	0 ± 0	0 ± 0	0 ± 0
3	**Ampicillin**	AMP 10	9.9 ± 0.2	6.5 ± 2.6	39 ± 4.0	0 ± 0	73.8 ± 8.4	9.4 ± 0.2	0 ± 0	0 ± 0	0 ± 0
40	**Chloramphenicol**	C 30	0 ± 0	9.7 ± 0.2	3.4 ± 2.7	0 ± 0	26.7 ± 3.15	0 ± 0	0 ± 0	0 ± 0	0 ± 0
5	**Aztreonam**	ATM 30	62.7 ± 4.6	31.2 ± 13.0	34 ± 3.8	35.81 ± 6.4	35.8 ± 5.5	27.2 ± 3.6	26.5 ± 0	31.33 ± 3.9	4.2 ± 3.5
6	**Amoxicillin clavulanic acid**	AMC 30	14.1 ± 0.2	4.9 ± 4.0	4.9 ± 4.0	54.3 ± 4.2	0 ± 0	7.3 ± 6.0	0 ± 0	0 ± 0	(−) 25.9 ± 0.4
7	**Gentamicin**	GEN 10	0 ± 0	(−) 51.7 ± 8.4	(−) 89.1 ± 0.6	0 ± 0	(−) 65.3 ± 3.9	(−) 65.3 ± 3.9	(−) 8.8 ± 7.2	(−) 8.8 ± 7.2	(−) 8.8 ± 7.2
8	**Ceftriaxone**	CRO 30	0 ± 0	26.4 ± 3.9	22.2 ± 0.5	0 ± 0	11.8 ± 0.1	47.1 ± 4.4	55.6 ± 9.3	32.9 ± 13.4	0 ± 0
9	**Ciprofloxacin**	CIP 5	(−) 55.3 ± 3.3	(−) 59.5 ± 3.9	(−) 59.5 ± 0	(−) 2.22 ± 1.8	(−) 14.5 ± 2.9	(−) 19.9 ± 2.9	(−) 65.3 ± 3.9	(−) 65.3 ± 3.9	(−) 65.3 ± 3.9

Data is reported as the mean ± SEM from three independent experiments

In case of *S. typhi*murium combining punicalagin at a sub-inhibitory concentration of 30 μg, the antibacterial efficacy of 30 μg of aztreonam was enhanced by 62%, and by further increasing the punicalagin to 100 μg and 500 μg, only showed 31 and 34% aztreonam augmenting efficacy (Table 4, Serial No. 5, Fig. S3). While for *S. typhi*, the combinations of 30 μg aztreonam /30, and 100 μg of punicalagin showed approximately equal inhibiting efficacy (Table 4, Serial No. 5, Fig. 6B). In case of *E. coli,* 30 μg and 100 μg punicalagin/30 μg aztreonam demonstrated 26 and 31% synergistic combinations, respectively. However, the 500 μg punicalagin/30 μg aztreonam combination proved to be less efficient with only 4% of synergism efficacy (Table 4, Serial No.5). The combination of 30 μg amoxicillin clavulanic acid/30 μg of punicalagin showed 14% antimicrobial augmenting potential for *S. typhi*murium but 30 g amoxicillin clavulanic acid/100 and 500 μg of punicalagin just showed 4.9% increment in the growth-inhibiting area (Table 4, Serial No. 6). For *S. typhi* punicalagin 30 μg and 500 μg demonstrated 54 and 7.3% amoxicillin clavulanic acid augmenting potential but 100 μg of punicalagin with 30 μg amoxicillin clavulanic acid remain indifferent combination against *S. typhi* (Table 4, Serial No. 6, Fig. 6C). However, 30 and 100 μg of punicalagin /30 μg amoxicillin clavulanic acid combination showed no effect on growth inhibiting area but 500 μg of punicalagin decreases the antimicrobial efficacy of 30 μg of amoxicillin clavulanic acid against *E. coli* Table 4, Serial No. 6)*.* Gentamicin 10 μg/ 30 μg punicalagin, the only combination that was ineffective against *S. typhi*murium while all other tested combinations of gentamicin 10 μg/ 30, 100 and 500 μg punicalagin against all of three clinical strains demonstrated antagonism by decreasing the growth inhibiting area (Table 4, Serial No. 7). Ceftriaxone 30 μg with punicalagin at concentration of 30 μg was ineffective combination for *S. typhi*murium while the combinations 30 μg ceftriaxone/100, 500-μg punicalagin was synergistic with the increase in growth inhibiting area by 24 and 22% **(**Table 4, Serial No. 8). The clinical strain *S. typhi* followed the same trend as 30 μg ceftriaxone/ 30 μg punicalagin showed no effect in growth inhibiting area but the combinations 30 μg ceftriaxone with 100 μg and 500 μg of punicalagin demonstrated synergism by increasing growth inhibiting zone by 11 and 47% (Table 4, Serial No. 8, Fig. 6D). However, *E. coli* was strongly inhibited by 55% with the synergistic combination of 30 μg ceftriaxone/ 30 μg punicalagin. The percentage synergistic inhibition (32%) decreased by increasing the punicalagin concentration to 100 μg while increasing the punicalagin concentration to 500 μg diminished the synergistic behavior (Table 4, Serial No. 8) at a concentration of 500 μg/30 μg ceftriaxone disc further enhanced the activity of ceftriaxone from 12 to 47% (Table 4, Serial No.8). The most antagonistic combination was observed with ciprofloxacin 5 μg/ 30, 100,500 μg punicalagin against all of three selected clinical strains (Table 4, Serial No.9).

The comparison of % increase or decrease in antibiotics activity is given in (Fig. 6, A-C). The synergistic efficacy of sub-inhibitory concentrations of punicalagin and antibiotics (previously observed by agar disc diffusion assay) were further evaluated by time-dependent growth curve assay. For plotting the time-response curves, the growth of *S. typhi*, *S. typhi*murium and *E. coli* cells in the presence of sub-inhibitory concentrations of punicalagin and sub-inhibitory concentrations of antimicrobials in combination and alone were monitored. OD_600_ was measured after an interval of 1 hour and up to 12 hours at 37 °C. Time-kill curves confirmed the bacteriostatic behaviour of punicalagin against exposed bacterial cells at sub-inhibitory concentrations. It was found that all the clinical strains in the panel showed less growth subjected to the simultaneous administration of sub-inhibitory concentrations of punicalagin and antimicrobials, compared with punicalagin and antibiotics alone. Moreover, no superimposition of graphs was noted at any point of data collection (Figs, 7, 8 and 9). The combination of Ampicillin/ punicalagin against *S. typhi* demonstrated the highest synergy while chloramphenicol/punicalagin against *S. typhi*murium proved to be the lowest synergistic combination.

**Fig. 7:**
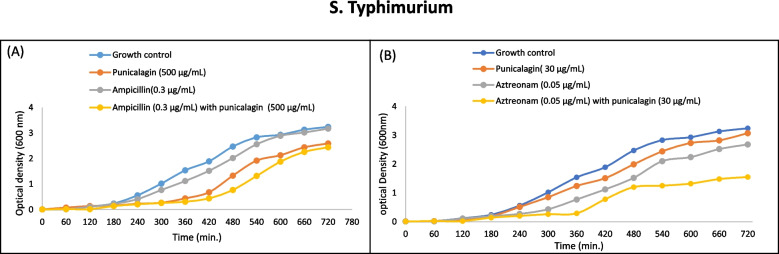
Comparative growth curves representing the drugs augmenting efficacy of punicalagin against *S. typhi*murium. **A** Time kill curve of *S. typhi*murium for ampicillin with and without punicalagin. Blue: Growth control no ampicillin/punicalagin; Red: punicalagin (500 μg/mL); Grey: Sub-inhibitory concentration of ampicillin (0.3 μg/mL); Yellow: Sub-inhibitory concentration of ampicillin (0.3 μg/mL) with punicalagin (500 μg/mL), (**B**) Time kill curves of *S. typhi*murium for aztreonam with and without punicalagin. Blue: Growth control, no aztreonam/punicalagin; Red: punicalagin (30 μg/mL); Grey: Sub-inhibitory concentration of aztreonam (0.05 μg/mL); Yellow sub-inhibitory concentration of aztreonam (0.05 μg/mL) with punicalagin (30 μg/mL)

**Fig. 8:**
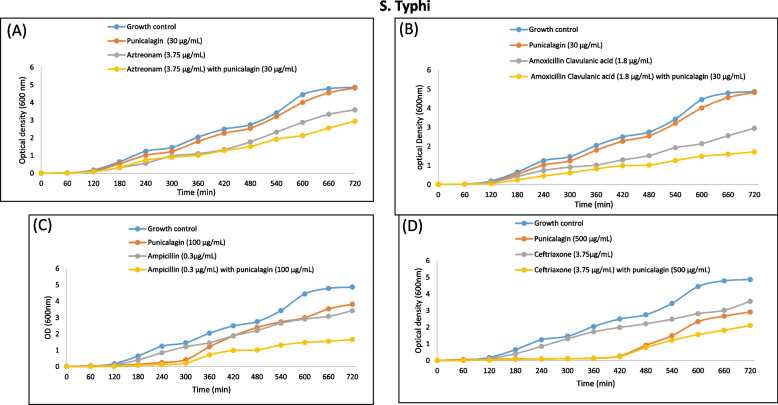
Comparative growth curves representing the drugs augmenting efficacy of punicalagin against *S. typhi*. **A** Time kill curves of *S. typhi* for Aztreonam with and without punicalagin. Blue: Growth control, no aztreonam/punicalagin; Red: punicalagin (30 μg/mL); Grey: Sub-inhibitory concentration of aztreonam (3.75 μg/mL); Yellow: Sub-inhibitory concentration of ATM (3.75 μg/mL) with punicalagin (30 μg/mL), (**B**) Time kill curves of *S. typhi* for amoxicillin clavulanic acid with and without punicalagin. Blue: Growth control, no amoxicillin clavulanic acid/punicalagin; Red; punicalagin (30 μg/mL); Grey: Sub-inhibitory concentration of amoxicillin clavulanic a (1.8 μg/mL); Yellow: sub-inhibitory concentration of amoxicillin clavulanic acid (1.8 μg/mL) with punicalagin (30 μg/mL), (**C**) Time kill curves of *S. typhi* for ampicillin with and without punicalagin. Blue: Growth control, no ampicillin/punicalagin; Red: punicalagin (100 μg/mL); Grey: Sub-inhibitory concentration of ampicillin (0.3 μg/mL); Yellow: sub-inhibitory concentration of ampicillin (0.3 μg/mL) with punicalagin (100 μg/mL), (**D**) Time kill curves of *S. typhi* for ceftriaxone with and without punicalagin. Blue: Growth control, no ceftriaxone/punicalagin; Red: punicalagin (500 μg/mL); Grey: Sub-inhibitory concentration of ceftriaxone (3.75 μg/mL); Yellow: sub-inhibitory concentration of ceftriaxone (3.75 μg/mL) with punicalagin (500 μg/mL)

**Fig. 9:**
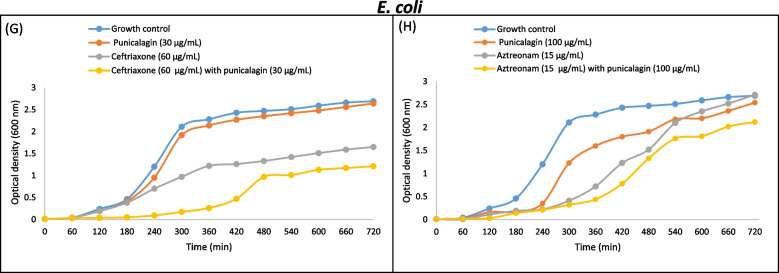
Comparative growth curves representing the drugs augmenting efficacy of punicalagin against *E. coli,* (**A**) Time kill curves of *E. coli* for ceftriaxone with and without punicalagin. Blue: Growth control, no ceftriaxone/punicalagin; Red: punicalagin (30 μg/mL); Grey: Sub-inhibitory concentration of ceftriaxone (60 μg/mL); Yellow: sub-inhibitory concentration of ceftriaxone (60 μg/mL) with sub-inhibitory concentration of punicalagin (30 μg/mL), (**B**) Time kill curves of *E. coli* for aztreonam with and without punicalagin. Blue: Growth control, no aztreonam/punicalagin; Red: punicalagin (15 μg/mL); Grey: Sub-inhibitory concentration of aztreonam (15 μg/mL); yellow: Sub-inhibitory concentration of aztreonam (15 μg/mL) with a sub-inhibitory concentration of punicalagin (100 μg/mL)

## Discussion

The selection of appropriate solvents for extraction plays a significant role in obtaining an acceptable yield of required compounds with good antimicrobial activity, which directly correlates with the polarity of solvents [44]. Extraction in 100% (v/v) concentration led to a less yield of plant metabolites, whereas a better yield was observed using a concentration of 70–80% (v/v) regardless of the solvent [44, 45]. Moreover, higher extraction yield does not correlate with high antimicrobial and antioxidant activity as it depends upon the extraction weightage of active compounds [46] also observed in this study that 80% pomegranate peel methanol extract was found antimicrobial with the highest activity against the tested clinical strains (Table 2). It has been observed that Gram-negative bacteria were more sensitive toward methanol peel extract than water extracts [47]. Moreover, a strong positive correlation was observed between the antibacterial efficacy of the 80% methanol pomegranate peel extracts with their calculated phenolic contents suggesting a higher extent of its bioactivity in polar solvents [48–50].

The inhibition zones against *E. coli, S. typhi* and *S. typhi*murium in our study were comparable to those of earlier studies, although the active concentrations (700 mg/mL stock solution, 100 μL/ well, 7000 μg/well) vary from the previously reported concentrations that were 800 μg/well – 12 mg/mL in earlier investigations [47, 51]. The difference in the activity of pomegranate extract among various studies could be explained based on the phenolic contents of the prepared extracts and pathogenic strain sensitivity [52]. Water extract and boiled water extracts both possessed only a weak activity or hazy zones against all tested bacteria at 12 h that disappeared upon prolonged incubation for 24–48 h. The disappearance of hazy zones and small zones may indicate that water extracts have a low activity or bacteriostatic nature due to the factor that the desired inhibition of the physiological processes of the microbes is overcome by pathogenic microbes upon prolonged exposure. Interestingly, ethyl acetate extracts were inactive for all tested organisms. These results are supported by the previous reports that plant extracts with non-polar solvents e.g., ethyl acetate, n-hexane and chloroform were inactive against pathogenic strains, making hydrophilic extractants an excellent choice for extracting bioactive polyphenolic constituents [49, 50, 52, 53].

Over the last 20 years, many studies reported the anti-*Enterobacteriaceae* efficacy of *P. granatum* extracts as have been compared in Table 5. Some comprehensive studies evaluated the anti-*Salmonella* activity of *P. granatum* peels, including against *S. typhi* and *S. typhi*murium, but the antibiotic resistance profile of *Salmonella* spp. was not determined (Table 5, Serial No.2,3,6,10,13), which have been pursued in the current study. In another study, non-probioticated as well as probioticated *P. granatum* juice was shown to be active against non-MDR *S. typhi* and *S. typhi*mrium (Table 5, Serial No.7). Anti-*E. coli* efficacy of *P. garanatm* crude extracts has been studied more rigorously than that of anti-*Salmonella* spp. Hydro-alcoholic extracts of *P. granatum* peel, juice, seeds and whole fruit showed good antimicrobial activity against *E.coli*, but mostly these studies had not determined the antibiotic resistance profile of used *E. coli* strains (Table 5, Serial No.1,4-7,10,11,13–15). Only a few studies considered the MDR strains of *E. coli* (Table 5, Serial No.18) evaluating the antimicrobial efficacy of *P. granatum* leaves only but in our study, the antimicrobial activity of all fruit parts of *P. garanatum* was compared against MDR *E. coli* strains showing resistance against third-generation cephalosporins and fluoroquinolones.

**Table 5:** Anti-*Enterobacteriaceae* activity of *Punica granatum* (2005–2022)

Sr. No.	Year	*P. granatum* part	Source	Concentration (mg/ml)	Bacteria	Antibiotic resistance profile	ZOI (mm)	MIC (mg/ml)	References
1	2005	Peel	Ethanol extract	2.5	*E. coli* (ATCC 25922)	ND^c^	10.9	0.39	[53]
					*E. coli* O157: H7		12.7	3.13	
					*E. coli* O26: H11		11.5	3.13	
					*E. coli* O111: NM		11.6	3.13	
					*E. coli* O22		12.0	1.56	
2	2008	Peel	Methanol extract	250 μg/mL	*S. typhi*	ND^c^	21.0	–	[54]
					*S. typhi*murium		25.0	–	
					*S. paratyphi* A		15.0	–	
					*S. paratyphi* B		18.0	–	
					*Sh. dysenteriae*		22.0	–	
					*Sh. flexneri*		25.0	–	
3	2008	Peel	Ethanol extract	500 μg/ml	*S.* Dublin ATCC 39184, *S. typhi* ATCC 19943, *S. paratyphi* A, *S. enteritidis,* *S. typhi*murium, *S.* Derby ATCC 6960, *S. gallinarum*	ND^c^	13.3 17.3 18.6 14.6 15.0 14.3 16.0	500 250 62.5 250 1000 500 250	[55]
					*S. choleraesuis* ATCC 7001	AM, SXT	16.0	62.5	
					*S. typhi*murium	AM, C, G, S, TIC	12.6	250	
					*S. gallinarum*	CTX, G, SXT	16.6	62.5	
					*S. gallinarum*	CTX, NA, CP	17.6	62.5	
					*S. gallinarum*	G, NA, S	16.3	125	
					*S. gallinarum* ATCC 9184	AMP, AMC, C, G, S	13.0	1000 μg/ml	
4	2009	Peel	Water ethanol extract	10	*E. coli (*ATCC 10536)	ND^c^	16.0	1.0	[51]
					*K. pneumoniae* ATCC 10031		16.0	2.0	
5	2010	Peel	Water methanol extract	60 μl	*E. coli* MTCC 732	ND^c^	20.0	–	[56]
		Seed red					11.0	–	
		Seed white					8.0	–	
		Whole fruit					15.0	–	
		Juice					17.0	–	
6	2011	Peel	Water methanol extract	5% (w/w) extract-based ointment	*S.* Anatum	ND^c^	–	0.25	[57]
					*S. typhi*murium		–	0.25	
					*E. coli* (ATCC 25922)		–	0.5	
7	2011	Juice	Non-probioticated	100 ml	*S. typhi* PTCC 1639	ND^c^	18.0	–	[58]
					*E. coli* ATCC 8739		20.0	–	
			Probioticated		*S. typhi* PTCC 1639		18.5	–	
					*E. coli* ATCC 8739		20.0	–	
8	2012	Peel	Methanol extract	1	*E. coli* (ATCC 35218)	ESBL producer	15.0		[59]
				10			15.0		
		Peel		1	*K. pneumoniae* (ATCC 700603)		18.0		
				10			12.0		
		Peel		10,240–5 μg/mL	*E. coli*	Third generation cephalosporin^a^ / Second generation fluoroquinolone^b^	–	640–2560 μg/ml	
					*K. pneumoniae*		–	1280–2560 μg/ml	
9	2012	Peel	Methanol extract	12.5	*E. coli* (ATCC 11775)	ND^c^	–	0.39–0.78	[60]
					*K. pneumoniae* (ATCC 13883)		–	0.20–0.39	
10	2014	Peel	Ethanol water extract	166.6	*K. pneumoniae* ATCC 10031	ND^c^	16.0	2.0	[33]
					*K. pneumoniae*		14.3		
			Water extract		*S. enterica* serovar Typhi		28.0		
11	2015	Peel	Ethanol extract	15	*E. coli*	ND^c^	32	–	[61]
		Seed		60			22	–	
12	2015	Peel	Methanol/Water extract	1024–0.5 μg/ml	*K. pneumoniae*	ESBL	- -	512- > 1024 μg/ml	[62]
						KPC			
13	2016	Peel	Water methanol extract	10–100	*E. coli*	ND^c^	–	50	[63]
					*Salmonella* spp.		–	50	
14	2018	Juice	–	10 g sugar + 12 ml pomegranate juice	*E. coli*	ND^c^	19.0	–	[64]
15	2021	Whole fruit	5% aqueous extract	250	*E. coli*	non-ESBL producers	12.0	–	[65]
16	2022	peel	Ethanol extract	1.5–3.0%	*S. enteritidis*	E, OX, NA, CL, AM, K, CTX, CP, T, AK, SXT, CZ, G, IPM	–	2.2 log CFU/g reduction	[66]
17	2022	Peel	Acetone extract	20 μl of (extract from 10 g powder)	*E. coli*	CTX, CEF, ATM	28.0	8 μg/ml	[32]
		Juice	Ethanol extract				19.0	128 μg/ml	
		Seed					12.0	256 μg/ml	
		Peel	Acetone extract		*K. pneumoniae*	ATM	20.0	128 μg/ml	
		Juice					23.0	16 μg/ml	
		Seed	Ethanol extract				14.0	256 μg/ml	
		Peel	Acetone extract		*Shigella* spp.	CTX, CEF, ATM	26.0	–	
		Juice	Ethanol extract				18.0	–	
		Seed					14.0	–	
18	2022	Leaves	Acetone water extract	5 to 0.04	*E. coli*	Penicillin	12.0	2.5	[27]
						CP /MDR	12.0	5	

*E. aerogenes Enterobacter aerogenes*: *S. typhi*murium: *Salmonella enterica* serovar Typhimurium*; S. gallinarum*: *Salmonella* Gallinarum*; S. typhi*: *Salmonella Typhi; S.* Dublin: *Salmonella* Dublin*; S.* Derby: *Salmonella* Derby; *S. choleraesuis*: *Salmonella Choleraesuis*; *S. enteritidis: Salmonella Enteritidis; S. paratyphi* A: *Salmonella Paratyphi* A; *S.* Anatum*: Salmonella* Anatum*; E. coli: Escherichia coli; Sh. dysenteriae*: *Shigella dysenteriae; Sh. flexneri*: *Shigella flexneri*; *K. pneumoniae*: *Klebsiella pneumonia*

*ATCC American Type Culture Collection*: *PTCC* Persian Type Culture Collection: *MDR*: Multi drug resistant: *ESBL* Extended spectrum β-lactamase

Erythromycin (E), Oxacillin (OX), Nalidixic acid (NA), Clindamycin (CL), Ampicillin (AM), Kanamycin (K), Ciprofloxacin (CP), Tetracycline (T), Amikacin (AK), Trimethoprim-sulfamethoxazole (SXT), Cefazolin (CZ), Gentamicin (G), Imipenem (IPM), Aztreonam (ATM), cephalothin (CEF), Cefotaxime (CTX), Ticarcillin (TIC), Streptomycin (S), Chloramphenicol (C), Cephalothin (CF), Sulfisoxazole (G)

^a^cefotaxime or ceftazidime/clavulanate (co-amoxiclav)

^b^Ciprofloxacin, Ofloxacin, Norfloxacin, Gatifloxacin

^c^Not determined

Several studies reported MICs of the hydroalcoholic peel extracts of pomegranate ranging from 0.39 to 50 mg/mL against *E. coli* and 0.25 to 50 mg/mL for *Salmonella* spp. supporting the results of the current study [47, 53, 57, 67]. The findings of the broth dilution assay revealed the values of MIC for pomegranate peel methanol extract were lower than those reported previously: 50 mg/mL for both *E. coli* and *Salmonella* spp. [63] while higher than those reported by Al-Zoreky as 1 mg/mL for *E. coli* and 4 mg/mL for *Salmonella Enteritidis* ATCC 4931 [51]. A range in the minimum inhibitory concentrations of pomegranate extracts among various studies could be explained based on different extraction conditions leading to the difference in MICs as *E. coli* MIC values range from 62.5–625 mg/mL with the change in extraction method [68]. Moreover, the fruit variety with its phenolic contents and targeted pathogenic strain sensitivity has a direct impact on inhibiting the efficacy of the prepared extracts [51, 52, 69].

The origin of fluoroquinolone resistance is predominantly the chromosomal mutations involving the modifications in target sites and variations in efflux pump expression rendering both processes the primary culprits of enhanced resistance in microbes [70]. *E. coli* has been reported to exhibit multidrug resistance because the AcrAB-TolC efflux system uses fluoroquinolone as the substrate [71]. Another study proposed a positive correlation between AcrA efflux system expression and enhanced resistance to ciprofloxacin [72]. Subsequently, natural bioactive polyphenolic compounds were evaluated and have been reported to act as efflux pump inhibitors leading to synergistically reversing the resistant nature of the microbes against the drugs [73]. Initially, it was reported that crude extract of pomegranat*e* peels may be an efflux inhibitor [74]. In contrast, Anam et al., 2019 proved that pomegranat*e* peel methanol extract showed no efflux pump inhibitory activity against *S. typhi* [75].. While the methanol extract of pomegranate serves as an efflux inhibitor in Gram-positive bacteria e.g., *S. aureus* RN-7044 as reported by Braga et al. [76].

Punicalagin and ellagic acid were reported as the major bioactive phenolic compounds in pomegranate peel powder [77–79]. However, in our study, punicalagin only showed hazy inhibition zones against targeted isolates which had been reduced upon prolonged incubation time but ellagic acid could not exert any inhibition zone, and was not active against the tested *Enterobacteriaceae* strains. Based on its antibacterial activity, punicalagin was selected for further evaluation of its co-activity with conventional antimicrobials against selected pathogens because a single chemical compound as a drug augmenting agent is preferable for further drug formulation rather than a crude herbal extract.

Punicalagin was the bioactive ellagitannin, detected by LCMS/MS in pomegranate peel methanol extract, that showed antimicrobial activity against all isolates as observed by agar well diffusion assay (Table 2, Serial No. 30, Fig. 4). However, the findings of MIC and growth curve assays demonstrated that punicalagin alone up to 10 mg/mL was unable to completely inhibit the growth of targeted bacteria in liquid cultures. Although the used dose significantly restricted the rate of reproduction of microbial cells or slow down the required microbiological process for normal growth at a certain level, albeit at a concentration higher than 10 mg/mL (Figs. 7, 8 and 9). In earlier studies, punicalagin has been reported to downregulate the quorum-sensing genes in *Salmonella* spp. at sub-inhibitory concentrations [80, 81] supporting the reduction of the total cell number of targeted pathogens due to compromised communication in the presence of punicalagin observed in this study. Punicalagin reduced the motility of *S. typhi*murium by affecting the flagellum-associated genes. The bacteriostatic efficacy of punicalagin may be attributed to reduced motility. As it was already proved that many plant extracts accede the motility reduction efficacy [82]. Moreover, the MDR pathogens may require a very high dose of punicalagin for bactericidal effects because of structural and genetic changes induced by mutations causing drug resistance. The MICs of punicalagin against non-MDR *Salmonella* spp. strains were observed in the range of 250–1000 μg/mL [81].

Although, in our study, punicalagin only disrupted the normal growth rate up to 10 mg/mL, it proved to be a powerful, concentration-dependent, sensitizing agent in combination with the tested drugs depending upon the specific bacteria.

Punicalagin enhances the efficiency of oxacillin against methicillin-resistant *S. aureus* as evaluated by checkerboard assay. Punicalagin has been reported to be a good potentiator to increase the efficacy of cefotaxime and oxacillin against Gram-positive bacteria by interfering with bacterial transcription mechanisms and as a virulence inhibitor [83, 84]. Whereas the Gram-negative bacteria possess an outer plasma membrane as a complex diffusional barrier, which can exert an additional resistance for many conventional drugs, making Gram-negative bacteria notably less sensitive as compared to Gram-positive bacteria. However, punicalagin has been reported to destabilize bacterial membranes, so membrane damage would likely allow greater absorption of antibiotics to toxic levels. Moreover, compromised efflux pumping causes lethal interactions making bacterial cells more sensitive to drugs that accelerate bacterial cell death [80, 85]. The sub-inhibitory concentrations of punicalagin were reported to decrease the *S. typhi*murium swarming ability and virulence factor expression as well. One of the noticeable characteristics of punicalagin is that it targets the AHL-dependent QS system directly involving its virulence, invasion and pathogenicity.

## Conclusions

In the current study, we have demonstrated that Pakistan-originated Kandhari pomegranate peel methanol extract exhibited antibacterial activity against all tested MDR clinical isolates. The results of ESI-MS/MS analysis together with antimicrobial assays revealed that a flavonoid, punicalagin, which is abundantly present in active pomegranate peel methanol extract could be an effective antimicrobial potentiating agent against resistant strains of *Enterobacteriacea*e. It showed antimicrobial sensitizing capabilities in a concentration-dependent manner when combined with the antimicrobials against the resistant strains. Our experimental data strongly suggest that drug boosting combinations are significant candidates for animal model testing and punicalagin, and may be explored in combination with currently available antimicrobials against highly resistant strains of *Enterobacteriaceae.* Moreover, there is a need of investigating the exact antimicrobial sensitizing mechanism of punicalagin.

### Supplementary Information

**Additional file 1.**

## Data Availability

All the important data generated or analysed during this study are included in this published article and its supplementary information files. Any additional data, if required, will be available from the corresponding author on request.

## Abbreviations

NIBGE-C: National Institute for Biotechnology and Genetic Engineering College
PIEAS: Pakistan Institute of Engineering and Applied Sciences
MDR: Multidrug resistance
MIC: Minimum inhibitory concentration
ESI: Electrospray ionization
PCR: Polymerase chain reaction
DNA: Deoxyribonucleic acid
LB: Lauria-Bertani
MF: McFarland
LC-MS: Liquid chromatography-mass spectrometry
PTFE: Polytetrafluoroethylene
HCl: Hydrochloric acid
ELISA: Enzyme-linked immunoassay

## References

[1] Abrar S, Hussain S, Khan RA, Ul Ain N, Haider H, Riaz S (2018). Prevalence of extended-spectrum-β-lactamase-producing *Enterobacteriaceae*: first systematic meta-analysis report from Pakistan. Antimicrob Resist Infect..

[2] Kang CI, Song JH (2013). Antimicrobial resistance in Asia: current epidemiology and clinical implications. Infect Chemother..

[3] Apanga PA, Ahmed J, Tanner W, Starcevich K, VanDerslice JA, Rehman U (2022). Carbapenem-resistant *Enterobacteriaceae* in sink drains of 40 healthcare facilities in Sindh, Pakistan: A cross-sectional study. PLoS One..

[4] Hadjadj L, Syed MA, Abbasi SA, Rolain J-M, Jamil B (2021). Diversity of carbapenem resistance mechanisms in clinical gram-negative bacteria in Pakistan. Microb Drug Resist..

[5] Qamar FN, Yousafzai MT, Khalid M, Kazi AM, Lohana H, Karim S (2018). Outbreak investigation of ceftriaxone-resistant *Salmonella enterica* serotype Typhi and its risk factors among the general population in Hyderabad, Pakistan: a matched case-control study. Lancet Infect Dis..

[6] Abrar S, Ain NU, Liaqat H, Hussain S, Rasheed F, Riaz S (2019). Distribution of Bla CTX− M, bla TEM, Bla SHV and Bla OXA genes in extended-spectrum-β-lactamase-producing clinical isolates: A three-year multi-center study from Lahore. Pakistan Antimicrob Resist Infect..

[7] Salehi B, Abu-Darwish MS, Tarawneh AH, Cabral C, Gadetskaya AV, Salgueiro L (2022). Antimicrobial resistance collaborators global burden of bacterial antimicrobial resistance in 2019: a systematic analysis. Lancet..

[8] Bayode MT, Olalemi AO, Oladejo BO (2021). Multiple antibiotic resistant index and detection of qnrS and qnrB genes in bacterial consortium of urine samples from clinical settings. Eur J Biol Res..

[9] Park SH, Byun JH, Choi SM, Lee DG, Kim SH, Kwon JC (2012). Molecular epidemiology of extended-spectrum beta-lactamase-producing *Escherichia coli* in the community and hospital in Korea: emergence of ST131 producing CTX-M-15. BMC Infect Dis..

[10] Raji MA, Jamal W, Ojemeh O, Rotimi VO (2015). Sequence analysis of genes mediating extended-spectrum beta-lactamase (ESBL) production in isolates of *Enterobacteriaceae* in a Lagos teaching hospital, Nigeria. BMC Infect Dis..

[11] Bilal H, Khan MN, Rehman T, Hameed MF, Yang X (2021). Antibiotic resistance in Pakistan: a systematic review of past decade. BMC Infect Dis..

[12] Bidell MR, Palchak M, Mohr J, Lodise TP (2016). Fluoroquinolone and third-generation-cephalosporin resistance among hospitalized patients with urinary tract infections due to *Escherichia coli:* do rates vary by hospital characteristics and geographic region?. Antimicrob Agents Chemother..

[13] Bhan MK, Bahl R, Bhatnagar S (2005). Typhoid and paratyphoid fever. Lancet..

[14] Karkey A, Thwaites GE, Baker S (2018). The evolution of antimicrobial resistance in *Salmonella Typhi*. Curr Opin Gastroenterol..

[15] Al Kraiem AA, Yang G, Al Kraiem F, Chen T (2018). Challenges associated with ceftriaxone resistance in *Salmonella*. Front Life Sci..

[16] Kim JH, Mogasale V, Im J, Ramani E, Marks F (2017). Updated estimates of typhoid fever burden in sub-Saharan Africa. Lancet Glob Health..

[17] Majowicz SE, Musto J, Scallan E, Angulo FJ, Kirk M, O'Brien SJ (2010). The global burden of nontyphoidal *Salmonella gastroenteritis*. Clin Infect Dis..

[18] Antunes P, Mourao J, Campos J, Peixe L (2016). Salmonellosis: the role of poultry meat. Clin Microbiol Infect..

[19] Nakatsuchi A, Inagaki M, Sugiyama M, Usui M, Asai T (2018). Association of *Salmonella* Serotypes with quinolone resistance in broilers. Food Saf (Tokyo)..

[20] Wajid M, Awan AB, Saleemi MK, Weinreich J, Schierack P, Sarwar Y (2019). Multiple drug resistance and virulence profiling of *Salmonella enterica* Serovars *typhimurium* and *Enteritidis* from poultry farms of Faisalabad. Pakistan Microb Drug Resist..

[21] Khameneh B, Diab R, Ghazvini K, Bazzaz BSF (2016). Breakthroughs in bacterial resistance mechanisms and the potential ways to combat them. Microb Pathog..

[22] Moloney MG (2016). Natural products as a source for novel antibiotics. Trends Pharmacol Sci..

[23] Chawla M, Verma J, Gupta R, Das B (2022). Antibiotic Potentiators against multidrug-resistant Bacteria: discovery, development, and clinical relevance. Front Microbiol..

[24] Jubair N, Rajagopal M, Chinnappan S, Abdullah NB, Fatima A (2021). Review on the antibacterial mechanism of plant-derived compounds against multidrug-resistant Bacteria (MDR). Evid Based Complement Alternat Med..

[25] Moga MA, Dimienescu OG, Bălan A, Dima L, Toma SI, Bîgiu NF (2021). Pharmacological and therapeutic properties of Punica granatum phytochemicals: possible roles in breast cancer. Molecules..

[26] Akhtar S, Ismail T, Fraternale D, Sestili P (2015). Pomegranate peel and peel extracts: chemistry and food features. Food Chem..

[27] Maphetu N, Unuofin JO, Masuku NP, Olisah C, Lebelo SL (2022). Medicinal uses, pharmacological activities, phytochemistry, and the molecular mechanisms of *Punica granatum L*. (pomegranate) plant extracts: A review. Biomed Pharmacother..

[28] Sharma K, Mahato N, Lee YR (2019). Extraction, characterization and biological activity of citrus flavonoids. Rev Chem Eng..

[29] Pirzadeh M, Caporaso N, Rauf A, Shariati MA, Yessimbekov Z, Khan MU (2021). Pomegranate as a source of bioactive constituents: A review on their characterization, properties and applications. Crit Rev Food Sci Nutr..

[30] Sharma A, Thakur N (2016). Influence of active packaging on quality attributes of dried wild pomegranate (*Punica granatum L.*) arils during storage. J Appl Nat Sci..

[31] Baldassarre F, Vergaro V, De Castro F, Biondo F, Suranna GP, Papadia P (2022). Enhanced bioactivity of pomegranate peel extract following controlled release from CaCO3 nanocrystals. Bioinorg Chem Appl..

[32] Debib A, Menadi S, Sahnouni F, Boukhatem MN, KACED A. (2022). Bacterial inhibitory effect of Algerian pomegranate (*Punica Granatum L*.) extracts (Peel, juice, and seed) against multidrug resistant Bacteria. Microbiol Biotechnol Food Sci..

[33] Malviya S, Arvind JA, Hettiarachchy N (2014). Antioxidant and antibacterial potential of pomegranate peel extracts. J Food Sci Technol..

[34] Moussa I, Gassem M, Al-Doss A, Sadik W, Mawgood AA (2010). Using molecular techniques for rapid detection of *Salmonella* serovars in frozen chicken and chicken products collected from Riyadh. Saudi Arabia Afr J Biotechnol..

[35] Saeed MA, Haque A, Ali A, Mohsin M, Bashir S, Tariq A (2009). A profile of drug resistance genes and integrons in *E. Coli* causing surgical wound infections in the Faisalabad region of Pakistan. J Antibiot (Tokyo)..

[36] Maniatis T, Fritsch E, Sambrook J (1989). Molecular cloning, manual cold spring, harbor laboratory.

[37] Liora M, Mihaiu M, Tăbăran A, Sd D, Iv C, Pivariu B (2013). Antimicrobial resistance evaluation of pathogen *Salmonella* strains isolated in pork and poultry meat. Bull Univ Agric Sci Vet Med Cluj Napoca..

[38] Humphries R, Bobenchik AM, Hindler JA, Schuetz AN (2021). Overview of changes to the clinical and laboratory standards institute performance standards for antimicrobial susceptibility testing. J Clin Microbiol..

[39] Mphahlele RR, Fawole OA, Makunga NP, Opara UL. Effect of drying on the bioactive compounds, antioxidant, antibacterial, and antityrosinase activities of pomegranate peel. BMC Complement Altern Med. 2016;16(1):143. 10.1186/s12906-016-1132-y PMC4881059 27229852

[40] Ahmad I, Beg AZ (2001). Antimicrobial and phytochemical studies on 45 Indian medicinal plants against multi-drug resistant human pathogens. J Ethnopharmacol..

[41] Andrews JM (2001). Determination of minimum inhibitory concentrations. J Antimicrob Chemother..

[42] Moghaddam KM, Iranshahi M, Yazdi MC, Shahverdi AR (2009). The combination effect of curcumin with different antibiotics against *Staphylococcus aureus*. Int J Green Pharm..

[43] Pillai SK, Moellering R, Eliopoulos GM (2005). Antimicrobial combinations. Antib Lab Med..

[44] Magangana TP, Makunga NP, Amos Fawole O, Opara UL (2021). Effect of solvent extraction and blanching pre-treatment on phytochemical, antioxidant properties, enzyme inactivation and antibacterial activities of ‘Wonderful’pomegranate peel extracts. Processes..

[45] Masci A, Coccia A, Lendaro E, Mosca L, Paolicelli P, Cesa S (2016). Evaluation of different extraction methods from pomegranate whole fruit or peels and the antioxidant and antiproliferative activity of the polyphenolic fraction. Food Chem..

[46] Singh M, Jha A, Kumar A, Hettiarachchy N, Rai AK, Sharma D (2014). Influence of the solvents on the extraction of major phenolic compounds (punicalagin, ellagic acid and gallic acid) and their antioxidant activities in pomegranate aril. J Food Sci Technol..

[47] Prashanth D, Asha MK, Amit A (2001). Antibacterial activity of *Punica granatum*. Fitoterapia..

[48] Shan B, Cai YZ, Brooks JD, Corke H (2007). The in vitro antibacterial activity of dietary spice and medicinal herb extracts. Int J Food Microbiol..

[49] Alzoreky N, Nakahara K (2003). Antibacterial activity of extracts from some edible plants commonly consumed in Asia. Int J Food Microbiol..

[50] Negi P, Jayaprakasha G (2003). Antioxidant and antibacterial activities of *Punica granatum* peel extracts. J Food Sci..

[51] Al-Zoreky NS (2009). Antimicrobial activity of pomegranate (*Punica granatum L*.) fruit peels. Int J Food Microbiol..

[52] Cowan MM (1999). Plant products as antimicrobial agents. Clin Microbiol Rev..

[53] Voravuthikunchai SP, Sririrak T, Limsuwan S, Supawita T, Iida T, Honda T (2005). Inhibitory effects of active compounds from Punica granatum pericarp on verocytotoxin production by enterohemorrhagic *Escherichia coli* O157: H7. J Health Sci..

[54] Pradeep B, Manojbabu M, Palaniswamy M (2008). Antibacterial activity of *Punica granatum L.* against gastrointestinal tract infection causing organisms. Ethnobot leafl..

[55] Choi JG, Kang OH, Lee YS, Chae HS, Oh YC, Brice OO (2011). In vitro and in vivo antibacterial activity of *Punica granatum* Peel ethanol extract against *Salmonella*. Evid Based Complement Alternat Med..

[56] Dahham SS, Ali MN, Tabassum H, Khan M (2010). Studies on antibacterial and antifungal activity of pomegranate (*Punica granatum L*.). Am Eurasian J Agric. Environ Sci..

[57] Hayouni EA, Miled K, Boubaker S, Bellasfar Z, Abedrabba M, Iwaski H (2011). Hydroalcoholic extract based-ointment from *Punica granatum L.* peels with enhanced in vivo healing potential on dermal wounds. Phytomed..

[58] Fazeli MR, Bahmani S, Jamalifar H, Samadi N (2011). Effect of probiotication on antioxidant and antibacterial activities of pomegranate juices from sour and sweet cultivars. Nat Prod Res..

[59] Dey D, Debnath S, Hazra S, Ghosh S, Ray R, Hazra B (2012). Pomegranate pericarp extract enhances the antibacterial activity of ciprofloxacin against extended-spectrum β-lactamase (ESBL) and metallo-β-lactamase (MBL) producing gram-negative bacilli. Food Chem Toxicol..

[60] Fawole OA, Makunga NP, Opara UL (2012). Antibacterial, antioxidant and tyrosinase-inhibition activities of pomegranate fruit peel methanol extract. BMC Complement Altern Med..

[61] Gaber A, Hassan MM, Dessoky E-DS, Attia AO. In vitro antimicrobial comparison of Taif and Egyptian pomegranate peels and seeds extracts. J Appl Biol Biotechnol. 2015;3(2):0–1.

[62] Dey D, Ray R, Hazra B (2015). Antimicrobial activity of pomegranate fruit constituents against drug-resistant *mycobacterium tuberculosis* and beta-lactamase producing *Klebsiella pneumoniae*. Pharm Biol..

[63] Gullon B, Pintado ME, Pérez-Álvarez JA, Viuda-Martos M (2016). Assessment of polyphenolic profile and antibacterial activity of pomegranate peel (*Punica granatum*) flour obtained from co-product of juice extraction. Food Cont..

[64] Shivsharan U, Ravva S (2018). Antimicrobial activity of pomegranate juice. Res J Pharm Technol..

[65] Daoutidou M, Plessas S, Alexopoulos A, Mantzourani I (2021). Assessment of antimicrobial activity of pomegranate, cranberry, and black chokeberry extracts against foodborne pathogens. Foods..

[66] Elafify M, Darwish WS, El-Toukhy M, Badawy BM, Mohamed RE, Shata RR (2022). Prevalence of multidrug resistant *Salmonella* spp. in dairy products with the evaluation of the inhibitory effects of ascorbic acid, pomegranate peel extract, and D-tryptophan against *Salmonella* growth in cheese. Int J Food Microbiol..

[67] Voravuthikunchai S, Lortheeranuwat A, Jeeju W, Sririrak T, Phongpaichit S, Supawita T (2004). Effective medicinal plants against enterohaemorrhagic *Escherichia coli* O157:H7. J Ethnopharmacol..

[68] Alexandre EMC, Silva S, Santos SAO, Silvestre AJD, Duarte MF, Saraiva JA (2019). Antimicrobial activity of pomegranate peel extracts performed by high pressure and enzymatic assisted extraction. Food Res Int..

[69] McCarrell EM, Gould SW, Fielder MD, Kelly AF, El Sankary W, Naughton DP (2008). Antimicrobial activities of pomegranate rind extracts: enhancement by addition of metal salts and vitamin C. BMC Complement Altern Med..

[70] Hooper DC (2001). Emerging mechanisms of fluoroquinolone resistance. Emerg Infect Dis..

[71] Yu EW, Aires JR, Nikaido H (2003). AcrB multidrug efflux pump of *Escherichia coli*: composite substrate-binding cavity of exceptional flexibility generates its extremely wide substrate specificity. J Bacteriol..

[72] Mazzariol A, Zuliani J, Cornaglia G, Rossolini GM, Fontana R (2002). AcrAB efflux system: expression and contribution to fluoroquinolone resistance in *Klebsiella* spp. Antimicrob Agents Chemother..

[73] Stavri M, Piddock LJ, Gibbons S (2007). Bacterial efflux pump inhibitors from natural sources. J Antimicrob Chemother..

[74] Jurenka JS (2008). Therapeutic applications of pomegranate (*Punica granatum L*.): a review. Altern Med Rev..

[75] Tariq A, Sana M, Shaheen A, Ismat F, Mahboob S, Rauf W (2019). Restraining the multidrug efflux transporter STY4874 of *Salmonella Typhi* by reserpine and plant extracts. Lett Appl Microbiol..

[76] Braga LC, Shupp JW, Cummings C, Jett M, Takahashi JA, Carmo LS (2005). Pomegranate extract inhibits Staphylococcus aureus growth and subsequent enterotoxin production. J Ethnopharmacol..

[77] Aqil F, Munagala R, Vadhanam MV, Kausar H, Jeyabalan J, Schultz DJ (2012). Anti-proliferative activity and protection against oxidative DNA damage by punicalagin isolated from pomegranate husk. Food Res Int..

[78] Wu D, Ma X, Tian W (2013). Pomegranate husk extract, punicalagin and ellagic acid inhibit fatty acid synthase and adipogenesis of 3T3-L1 adipocyte. J Funct Foods..

[79] Nuncio-Jauregui N, Nowicka P, Munera-Picazo S, Hernández F, Carbonell-Barrachina ÁA, Wojdyło A (2015). Identification and quantification of major derivatives of ellagic acid and antioxidant properties of thinning and ripe Spanish pomegranates. J Funct Foods..

[80] Li G, Xu Y, Pan L, Xia X (2020). Punicalagin damages the membrane of *Salmonella typhimurium*. J Food Prot..

[81] Li G, Yan C, Xu Y, Feng Y, Wu Q, Lv X (2014). Punicalagin inhibits *Salmonella* virulence factors and has anti-quorum-sensing potential. Appl Environ Microbiol..

[82] Inamuco J, Veenendaal AK, Burt SA, Post JA, Tjeerdsma-van Bokhoven JL, Haagsman HP (2012). Sub-lethal levels of carvacrol reduce *Salmonella typhimurium* motility and invasion of porcine epithelial cells. Vet Microbiol..

[83] Mun SH, Kang OH, Kong R, Zhou T, Kim SA, Shin DW (2018). Punicalagin suppresses methicillin resistance of *Staphylococcus aureus* to oxacillin. J Pharmacol Sci..

[84] Song W, Wang L, Jin M, Guo X, Wang X, Guan J (2022). Punicalagin, an inhibitor of Sortase A, is a promising therapeutic drug to combat methicillin-resistant *Staphylococcus aureus* infections. Antimicrob Agents Chemother..

[85] Chusri S, Villanueva I, Voravuthikunchai SP, Davies J (2009). Enhancing antibiotic activity: a strategy to control *Acinetobacter* infections. J Antimicrob Chemother..

