# Supplementary material for: Punicalagin, a pomegranate polyphenol sensitizes the activity of antibiotics against three MDR pathogens of the Enterobacteriaceae
Source: BMC Complement Med Ther. 2024 Feb 16;24:93. doi: 10.1186/s12906-024-04376-7 (PMC10870630; doi:10.1186/s12906-024-04376-7)
Supplement: Supplementary file 1 — Additional file 1. [file 12906_2024_4376_MOESM1_ESM.docx]

**Supplementary Materials**

**Journal:** BMC Complementary Medicine and Therapies

**Title:** Punicalagin, a pomegranate polyphenol sensitizes the activity of antibiotics against MDR pathogens of the *Enterobacteriaceae*.

Saba Kiran^1^, Anam Tariq^1^, Zubera Naseem^1^, Sobia Jabeen^1^, Waqar Siddique^1^, Sobia Jabeen^1^, Shoaib Iqbal^1^, Rizwan Bashir^1^, Moaz-ur-Rehman^2^, Fazl-e-Habib^1^, Waqar Rauf^1^*, Aamir Ali^1^, Yasra Sarwar^1^, Georg Jander^3^, Mazhar Iqbal^1^*

^1^Health Biotechnology Division, National Institute for Biotechnology and Genetic Engineering College, Pakistan Institute of Engineering and Applied Sciences (NIBGE-C, PIEAS), Faisalabad-38000, Punjab, Pakistan.

^2^School of Biological Sciences, University of the Punjab, Quaid-i-Azam campus, Lahore, 54810, Punjab, Pakistan.

^3^Boyce Thompson Institute, Cornell University, Ithaca, 14850, New York, USA.

**Corresponding authors:** Mazhar Iqbal, PhD, Professor & Head, Health Biotechnology Division, National Institute for Biotechnology and Genetic Engineering College, Pakistan Institute of Engineering and Applied Sciences (NIBGE-C, PIEAS), Jhang Road, Faisalabad 38000, Punjab, Pakistan. Email: [hamzamgondal@gmail.com](mailto:hamzamgondal@gmail.com).

**Supplementary Figures:**

**
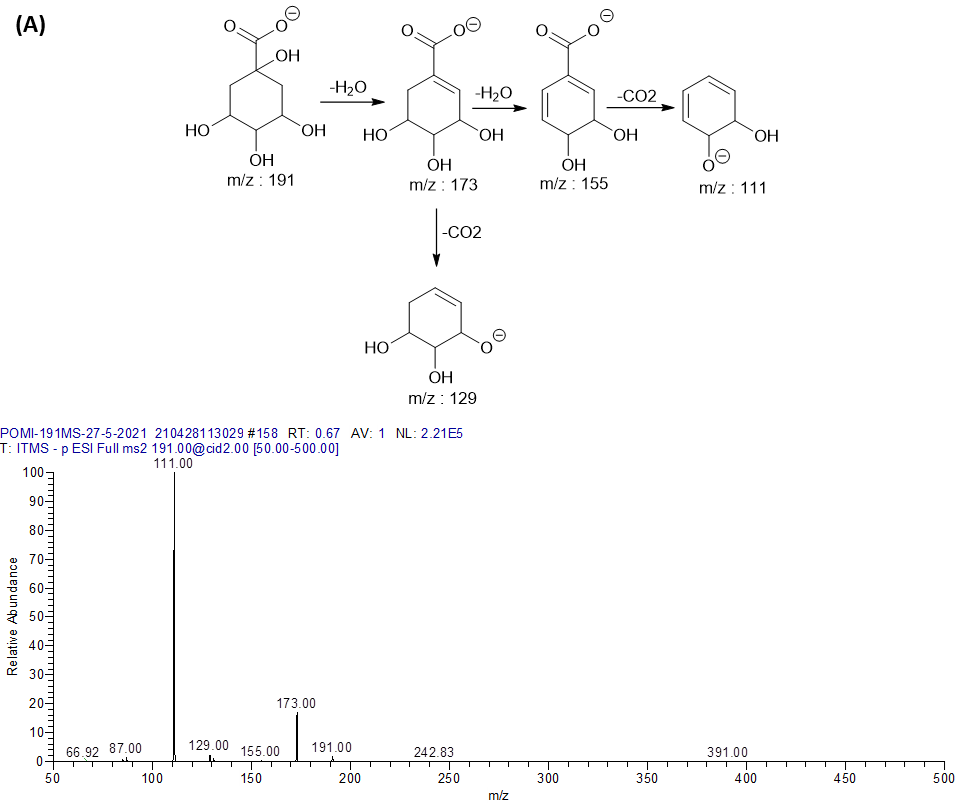
**

**Figure S1 (A):** Proposed fragmentation of quinic acid generated through quasi-ESI-MS^n^ in negative ion mode.

**
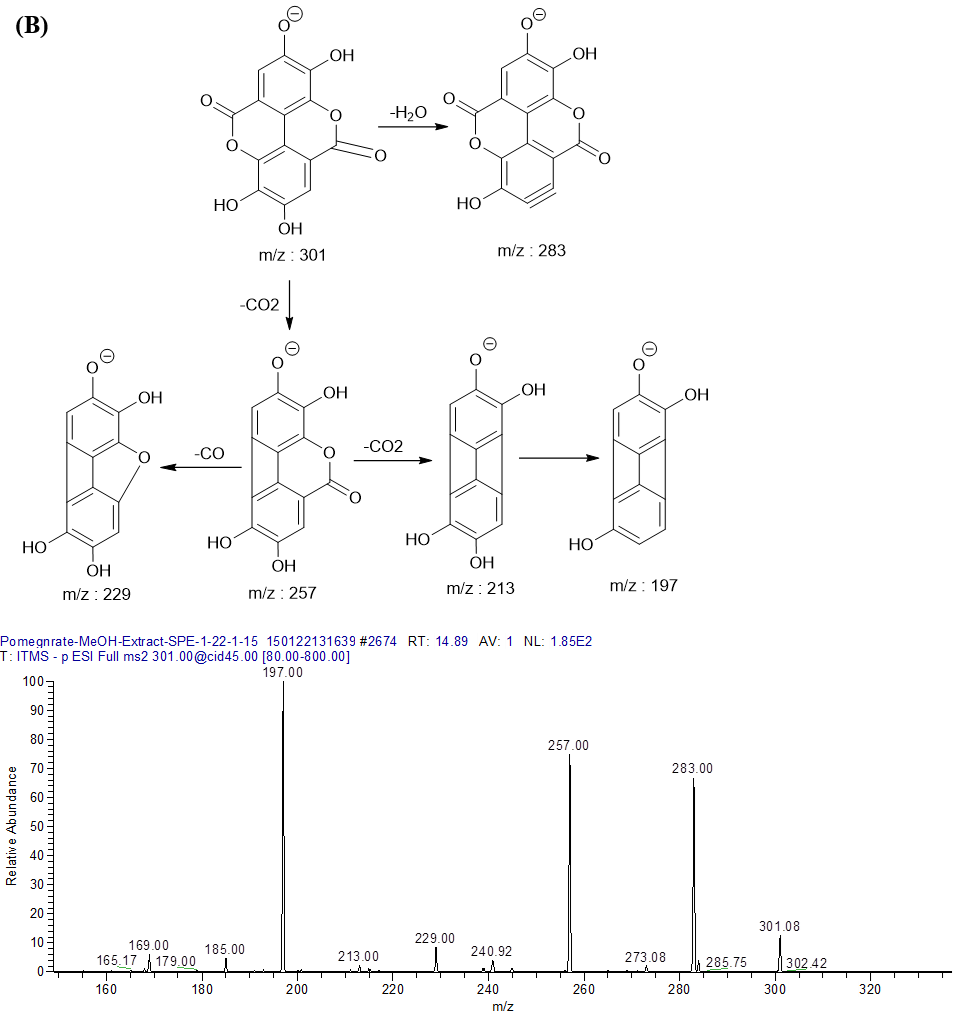
**

**Figure S1 (B):** Proposed fragmentation of ellagic acid generated through quasi-ESI-MS^n^ in negative ion mode.

**
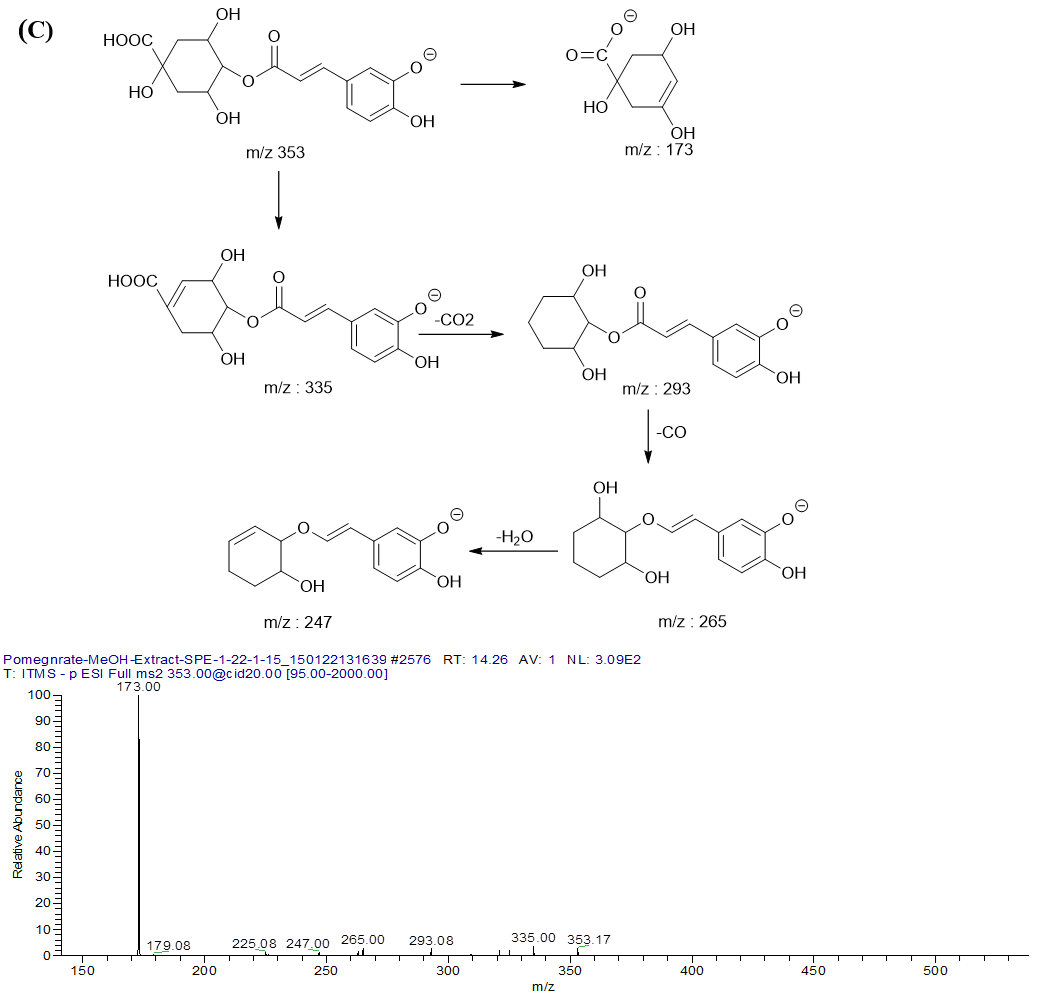
**

**Figure S1 (C):** Proposed fragmentation of cryptochlorogenic acid generated through quasi-ESI-MS^n^ in negative ion mode.

**
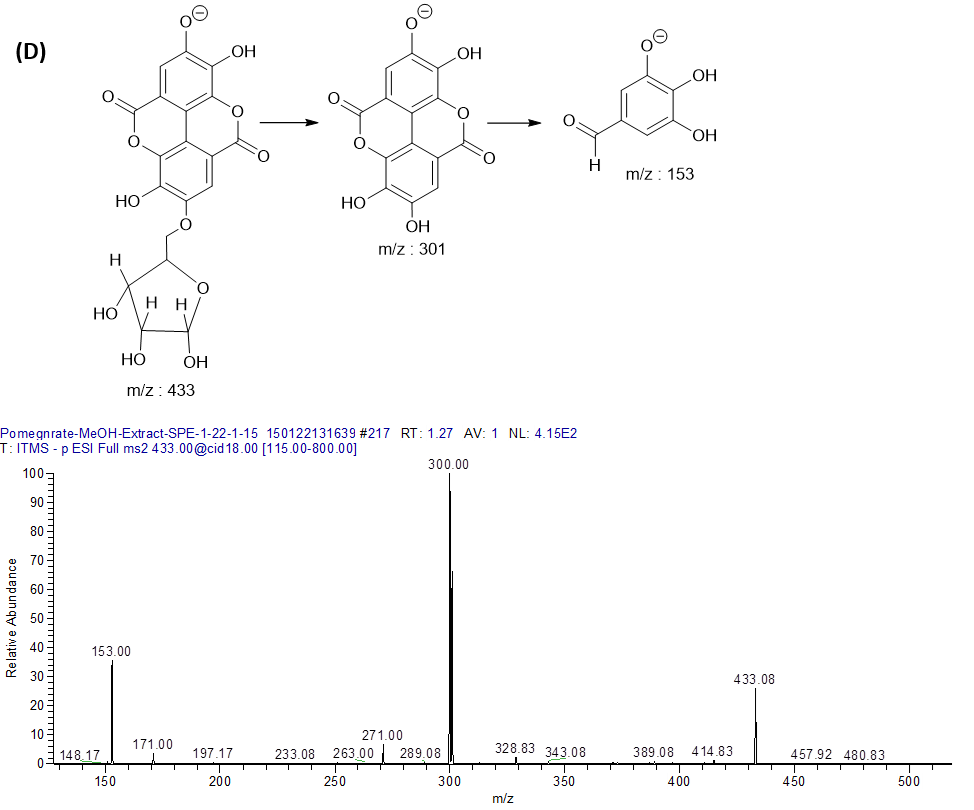
**

**Figure S1 (D):** Proposed fragmentation of ellagic acid pentoside generated through quasi-ESI-MS^n^ in negative ion mode.

**
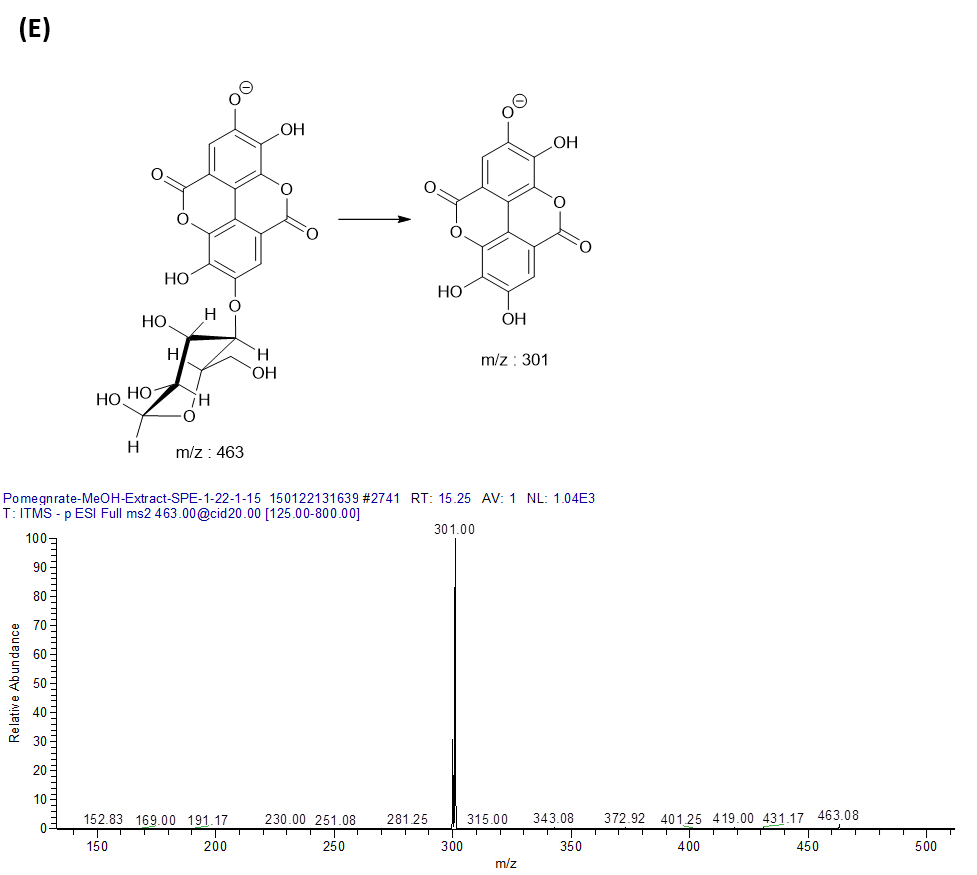
**

**Figure S1 (E)**: Proposed fragmentation of ellagic acid hexoside generated through quasi-ESI-MS^n^ in negative ion mode.

**
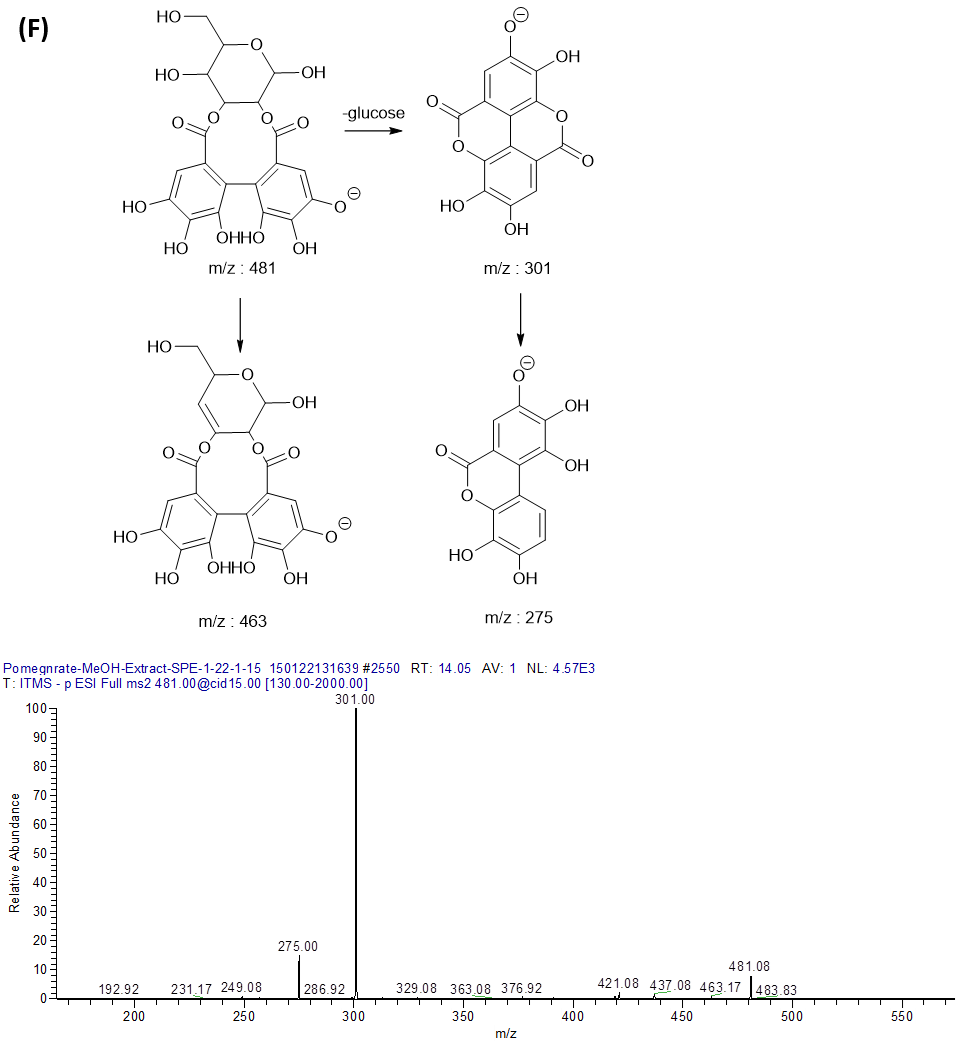
**

**Figure S1 (F):** Proposed fragmentation of pedunculagin fragment generated through quasi-ESI-MS^n^ in negative ion mode.

**
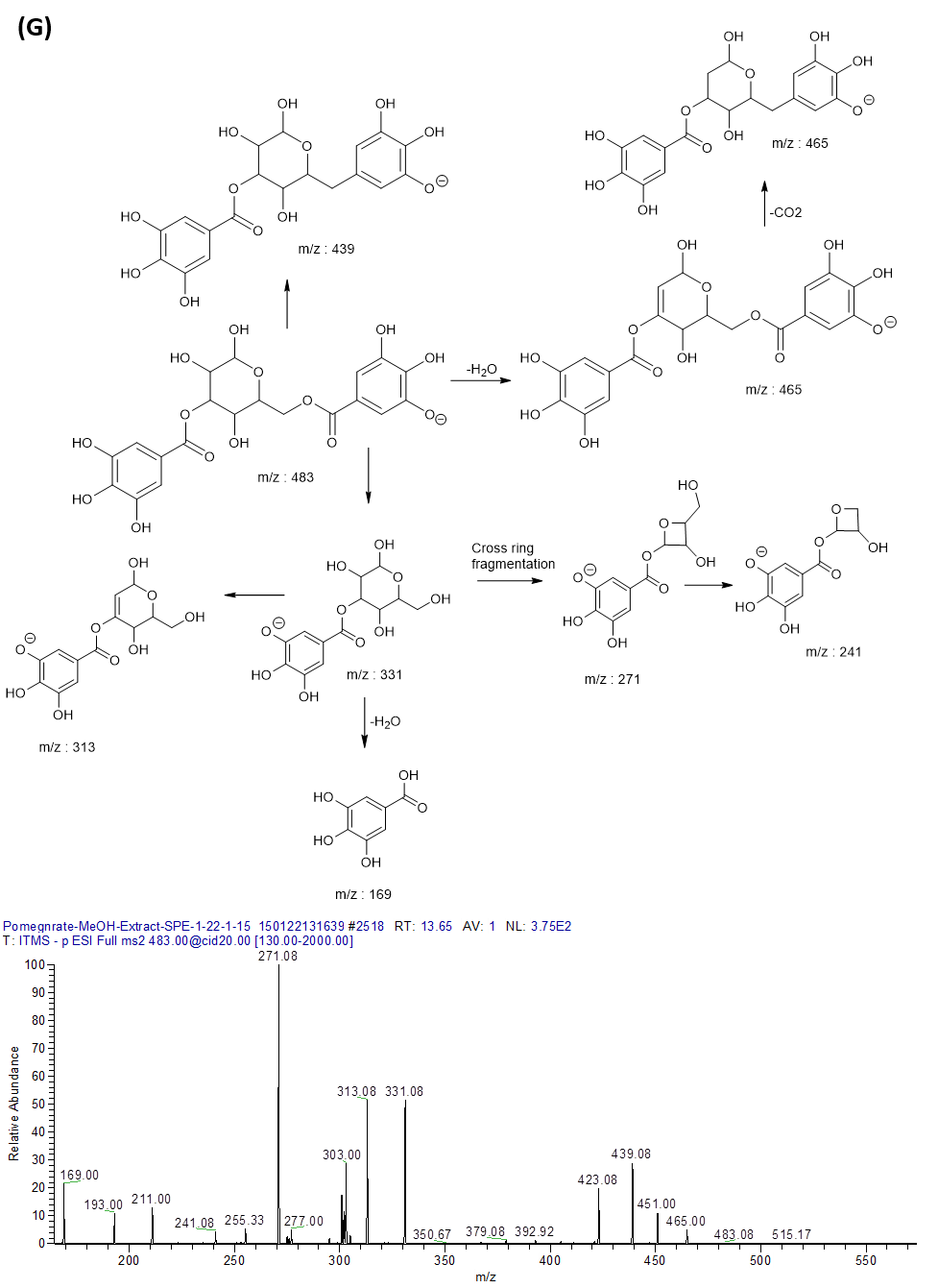
**

**Figure S1 (G):** Proposed fragmentation of digalloyl-glucose isomer generated through quasi-ESI-MS^n^ in negative ion mode.

**
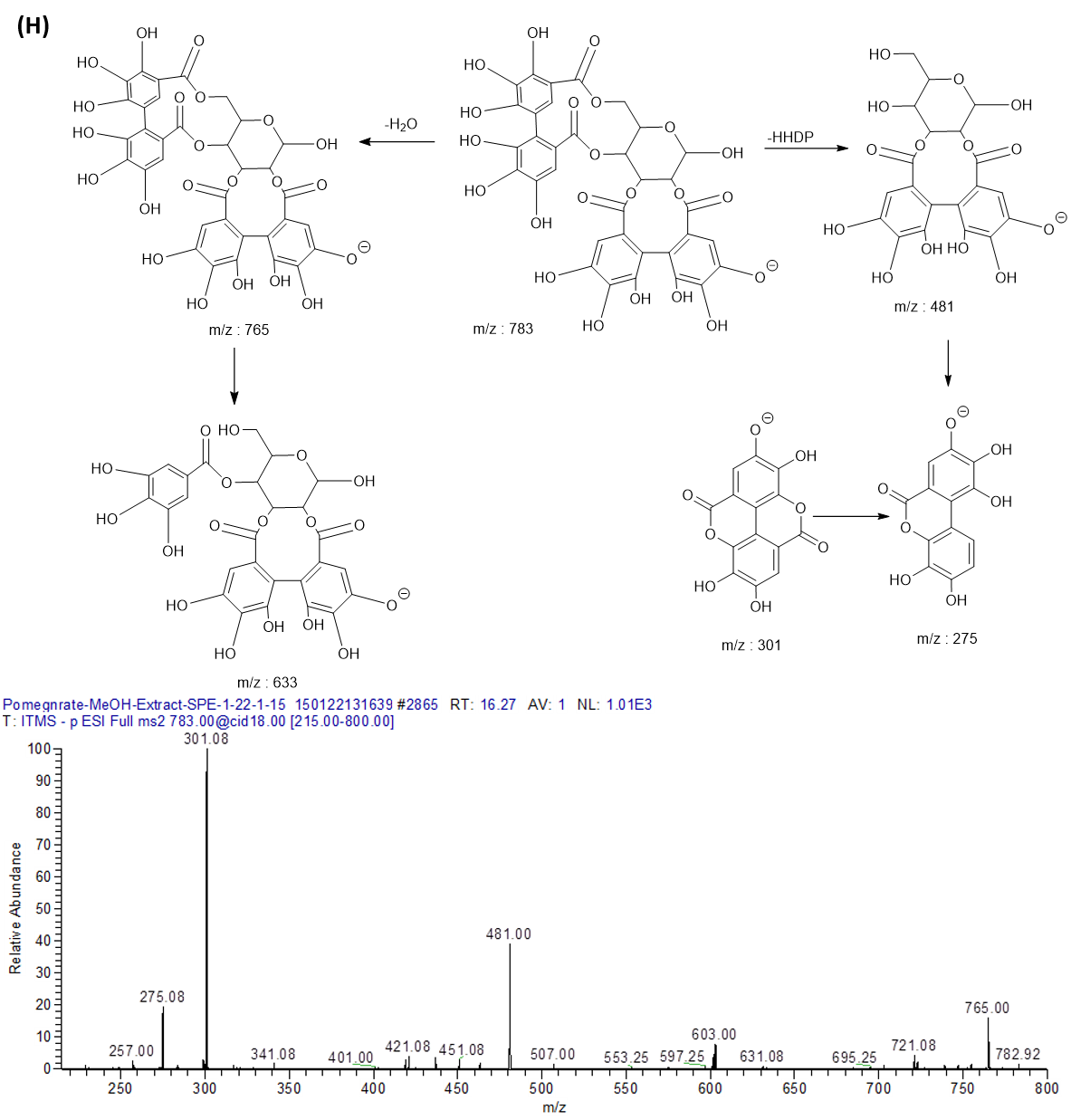
**

**Figure S1 (H):** Proposed fragmentation of pedunculagin generated through quasi-ESI-MS^n^ in negative ion mode.

**
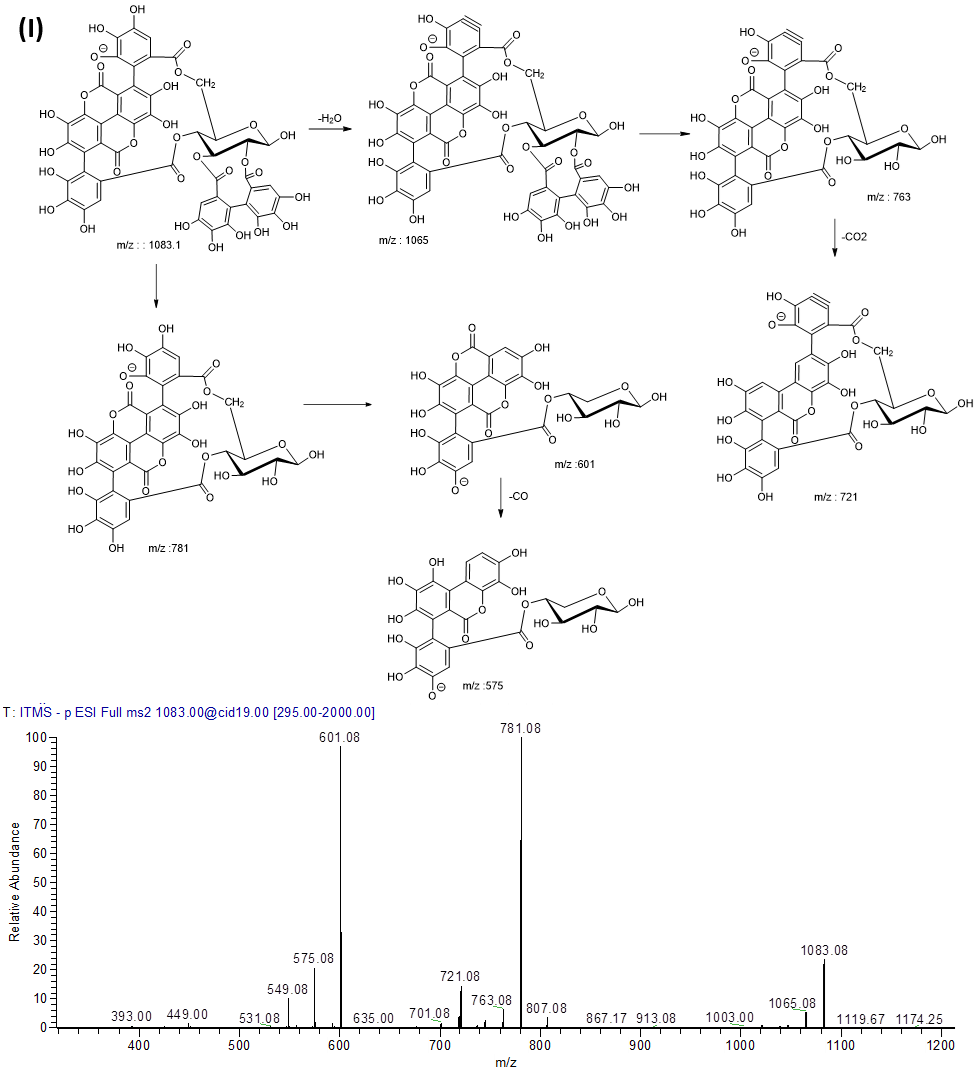
**

**Figure S1 (I):** Proposed fragmentation of punicalagin generated through quasi-ESI-MS^n^ in negative ion mode.

**
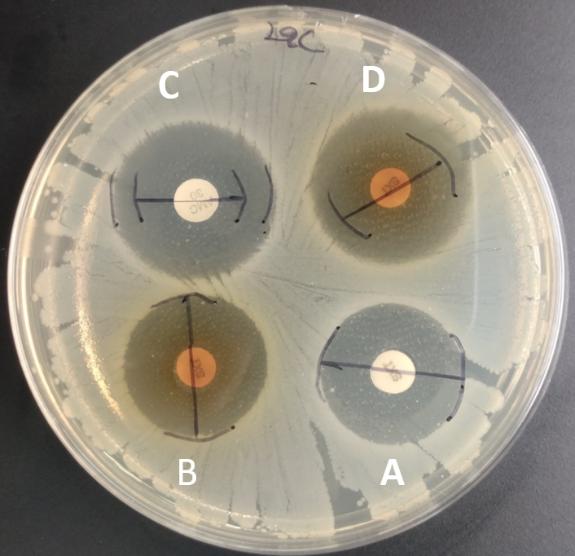
**

**Figure S2:** Combination effect of punicalagin with Sulfamethoxazole/Trimethoprim 23/1.25 µg against *S*. Typhi, (A) Inhibition zone produced by sulfamethoxazole/Trimethoprim 23/1.25 µg without punicalagin, (B) Combination effect of 100 µg punicalagin with sulfamethoxazole/Trimethoprim 23/1.25 µg (SXT-23/1.25) against MDR *S*. Typhi, (C) Amoxicillin clavulanic acid 30 µg (AMC 30) as a positive control, (D) Synergistic combination effect of 500 µg punicalagin with sulfamethoxazole/Trimethoprim 23/1.25 µg (SXT-23/1.25) against MDR *S*. Typhi.

**
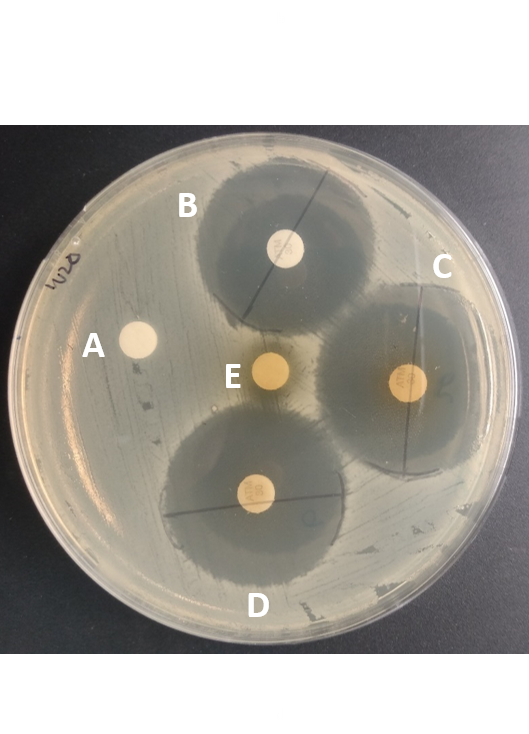
**

**Figure S3:** Combination effect of punicalagin with aztreonam 30 µg (ATM-30 µg) against *S*.Typhimurium, **(A)** Methanol as a negative control, **(B)** Inhibition zone produced by ATM-30 µg without punicalagin, **(C)** Synergistic combination effect demonstrated by a combination of 30 µg punicalagin with ATM-30 µg, **(D)** Indifferent combination effect demonstrated by a combination of 30 µg punicalagin with ATM-30 µg, **(E)** 500 µg punicalagin showed no inhibitory effect.

**Supplementary Tables:**

Table S1: Oligonucleotides used for confirmation of clinical isolates

| Bacteria | Genes | Primer sequences (5^/^-3^/^) | | Amplicon size (bp) | References |
| --- | --- | --- | --- | --- | --- |
| *E. coli* | *uidA* | F  R | ATCACCGTGGTGACGCATGTCGC  CACCACGATGCCATGTTCATCTGC | 486 | ([1](#_ENREF_1)) |
| *S.* Typhi | *fliC* | F  R | TATGCCGCTACATATGATGAG  TTAACGCAGTAAAGAGAG | 495 | ([2](#_ENREF_2)) |
| *S.* Typhimurium | *stm* | F  R | TTGTTCACTTTTTACCCCTGAA  CCCTGACAGCCGTTAGATATT | 401 | ([3](#_ENREF_3)) |
| Genus *Salmonella* | *invA* | F  R | GTGAAATTATCGCCACGTTCGGGCAA  TCATCGCACCGTCAAAGGAACC | 284 | ([4](#_ENREF_4)) |

**Supplementary references:**

1. Heininger A, Binder M, Schmidt S, Unertl K, Botzenhart K, Doring G. PCR and blood culture for detection of *Escherichia coli* bacteremia in rats. J Clin Microbiol. 1999;37(8):2479-82.

2. Song JH, Cho H, Park MY, Na DS, Moon HB, Pai CH. Detection of *Salmonella typhi* in the blood of patients with typhoid fever by polymerase chain reaction. J Clin Microbiol. 1993;31(6):1439-43.

3. Liora M, Mihaiu M, Tabaran A, Dan SD, Cordis IV, Pivariu B, et al. Antimicrobial Resistance Evaluation Napoca. 2013;70(2):266-70.

4. Moussa I, Gassem M, Al-Doss A, Sadik W, Mawgood AA. Using molecular techniques for rapid detection of *Salmonella* serovars in frozen chicken and chicken products collected from Riyadh, Saudi Arabia. Afr J Biotechnol. 2010;9(5):612-9.
